# UAV-based estimation of sunflower leaf flavonol content across multiple flight heights using RGB–multispectral canopy features and machine learning

**DOI:** 10.3389/fpls.2026.1931472

**Published:** 2026-08-31

**Authors:** Guotao Han, Jing Zhao, Zhida Song, Zihan Zhang, Liqun Lu, Jian Yuan

**Affiliations:** 1 School of Agricultural Engineering and Food Science, Shandong University of Technology, Zibo, China; 2 School of Transportation and Vehicle Engineering, Shandong University of Technology, Zibo, China

**Keywords:** feature selection, flavonol content, grey wolf optimizer, sunflower, transformer, UAV remote sensing

## Abstract

Rapid and non-destructive estimation of leaf flavonol content (Flav) is important for assessing crop physiological status and stress response. In this study, unmanned aerial vehicle (UAV)-based red, green, and blue (RGB) and multispectral imagery was used to estimate sunflower leaf Flav across the seedling, budding, flowering, and maturity stages. Data were collected at a single experimental site in the Yellow River Delta, China, during the 2025 growing season. UAV images were acquired at flight heights of 30 m, 50 m, and 80 m, and ground measurements of Flav were obtained within the same acquisition window using an MPM-100 plant multispectral pigment meter. A total of 320 plot-level observations were obtained from 80 sampling plots, which were divided at the plot level into 56 training plots and 24 testing plots, with all four growth-stage observations from the same plot assigned to the same subset. Feature selection and model development were conducted using the training data, which were further divided for hyperparameter optimization, whereas the testing set was used for comparative performance evaluation. A total of 44 UAV-derived features, including 22 vegetation indices, 16 texture features, and six color features, were extracted from sunflower canopy imagery. Pearson correlation coefficient (PCC) analysis and the Boruta algorithm were combined to identify compact feature subsets, and six regression models, including K-nearest neighbors, random forest, CatBoost, support vector regression, LightGBM, and Transformer regression, were evaluated. Genetic algorithm (GA), particle swarm optimization (PSO), and grey wolf optimizer (GWO) were further used to tune the Transformer model at the intermediate flight height of 50 m. The PCC-Boruta strategy reduced feature dimensionality and produced favorable predictive performance on the testing set. Among the evaluated regression models, the Transformer model achieved the best overall performance. Using the optimal feature combination, the PCC-Boruta-Transformer model obtained test-set R^2^ values of 0.932, 0.911, and 0.909 at 30 m, 50 m, and 80 m, respectively, with corresponding root mean square error (RMSE) values of 0.060, 0.069, and 0.070. Thus, the 30 m model provided the highest predictive accuracy among the three unoptimized flight-height models. The 50 m dataset, representing the intermediate level among the three evaluated flight heights, was used for subsequent hyperparameter optimization. Among the evaluated optimization configurations, the GWO-tuned Transformer achieved the best observed testing-set performance, with an R^2^ of 0.947 and an RMSE of 0.053. These results demonstrate the feasibility of the evaluated workflow under the specific site, growing-season, sensor, and management conditions of this experiment.

## 1 Introduction

Plant pigments are important biochemical indicators of crop physiological status, canopy function, and stress response. Among these pigments, flavonols are polyphenolic compounds involved in plant adaptation to environmental stress and are closely associated with crop growth regulation and nutrient status (Jia et al., 2015; Wang et al., 2024). Leaf flavonol content (Flav) tends to increase under nitrogen deficiency and other stress conditions, making it a useful indicator for evaluating crop growth and field management status (Padilla et al., 2018; Huang et al., 2019; Karaca et al., 2023; Vatter et al., 2024). Compared with chlorophyll-related measurements, Flav measurements are less affected by short-term illumination variation and can provide relatively stable information for crop physiological assessment. However, conventional Flav measurement methods generally require manual leaf sampling or contact-based measurements, which are labor-intensive, time-consuming, and difficult to apply for continuous monitoring at the field scale. Therefore, developing a rapid, non-destructive, and accurate method for estimating sunflower leaf Flav is important for crop growth monitoring, precision water and fertilizer management, and yield-relevant physiological assessment.

Remote sensing provides an effective technical pathway for non-destructive crop pigment monitoring because it can rapidly capture canopy spectral, structural, and spatial information over large field areas (Du et al., 2020; Pan et al., 2021; Xu et al., 2023). In recent years, unmanned aerial vehicle (UAV) platforms equipped with visible, multispectral, hyperspectral, and thermal sensors have been widely used for crop growth assessment, pigment estimation, leaf area index monitoring, and stress diagnosis (Tahir et al., 2018; Prudente et al., 2021; Li et al., 2024; Zhang et al., 2024; Khan et al., 2024; Liu et al., 2024). Vegetation indices derived from UAV imagery can reflect canopy greenness, photosynthetic activity, and physiological variation and have therefore become common predictors for crop biochemical parameter estimation. Nevertheless, relying only on vegetation indices may limit model performance, especially under high canopy coverage, where index saturation and background interference can reduce sensitivity to subtle physiological changes (Ni-Meister et al., 2018). To overcome this limitation, increasing attention has been paid to multi-feature fusion strategies that combine vegetation indices, color features, and texture features. Such feature combinations can describe canopy spectral response, visual color variation, and spatial heterogeneity more comprehensively, thereby improving the estimation of crop pigment-related traits.

UAV flight height is another key factor affecting crop parameter estimation because it directly influences spatial resolution, image coverage, data acquisition efficiency, and the extraction quality of canopy features (Stobbelaar et al., 2022; Mao et al., 2023; Yang et al., 2024). Lower flight heights usually provide higher spatial resolution, allowing finer canopy details such as leaf condition, crop density, and local heterogeneity to be captured. However, low-altitude flights also reduce image coverage efficiency and may increase sensitivity to field environmental disturbance (Luo et al., 2022). Higher flight heights can improve operational efficiency and reduce data acquisition time, but the decreased spatial resolution may weaken the representation of small-scale canopy spectral and textural differences (Xie and Yang, 2020). Previous studies have demonstrated that flight height can significantly influence the estimation accuracy of crop physiological and structural parameters. For example, UAV multispectral imagery has been used to estimate wheat soil plant analysis development (SPAD) values at different growth stages and flight heights, while multi-source UAV imagery has also been applied to maize leaf area index estimation across multiple flight heights (Yin et al., 2023; Liu et al., 2023). These studies indicate that determining an appropriate UAV flight height is essential for balancing estimation accuracy and field data acquisition efficiency.

Machine learning has become a powerful tool for crop biochemical and physiological parameter estimation because it can model complex nonlinear relationships between remote sensing features and ground-measured crop traits. Algorithms such as random forest, support vector regression, gradient boosting models, and deep learning methods have shown strong potential in crop pigment and growth parameter estimation (Li and Huang, 2021; Jiang et al., 2023; Tian et al., 2024; Nian et al., 2024; Wang et al., 2021; Sudu et al., 2022). However, UAV-based crop monitoring datasets often contain redundant features, noise, multicollinearity, and limited sample sizes, which may reduce model robustness and generalization. Feature selection methods can reduce input dimensionality, identify sensitive variables, and improve model interpretability. Pearson correlation coefficient (PCC) analysis is useful for selecting features with strong linear relationships with target variables, whereas the Boruta algorithm can evaluate feature importance based on random forest and capture nonlinear feature contributions. Combining these two methods may provide a more reliable feature subset by integrating linear correlation and model-based importance.

Although UAV remote sensing has been increasingly applied to crop growth monitoring, field-scale estimation of sunflower leaf Flav remains insufficiently explored. In particular, the combined effects of UAV flight height, fusion of red, green, and blue (RGB) and multispectral canopy features, feature-selection strategies, and regression models have rarely been evaluated systematically under field conditions. To address this gap, this study established a sequential UAV-based estimation workflow. First, RGB and multispectral imagery acquired at 30 m, 50 m, and 80 m were used to extract vegetation indices, color features, and texture features. Second, PCC and Boruta were applied individually and jointly to reduce feature redundancy and identify compact predictor subsets. Third, six regression models were compared to evaluate their ability to model the relationships between UAV-derived features and sunflower leaf Flav. Finally, genetic algorithm (GA), particle swarm optimization (PSO), and grey wolf optimizer (GWO) were evaluated for Transformer hyperparameter optimization at the intermediate flight height of 50 m. Specifically, the following research questions were addressed:

1. How does UAV flight height affect model predictive performance?
2. How do different combinations of vegetation indices, color features, and texture features affect predictive performance?
3. How does the predictive performance of the combined PCC-Boruta feature-selection strategy compare with that of PCC or Boruta alone across different regression algorithms and feature sets?
4. Under identical data partitions and optimization settings, does the GWO-tuned Transformer outperform the GA- and PSO-tuned Transformer models?

The contribution of this study lies in the integrated field-scale evaluation of these acquisition, feature-processing, and modeling components in a single-site and single-growing-season experiment, rather than in the development of a new optimization algorithm or Transformer architecture.

## 2 Materials and methods

### 2.1 Study area

**Figure 1:**
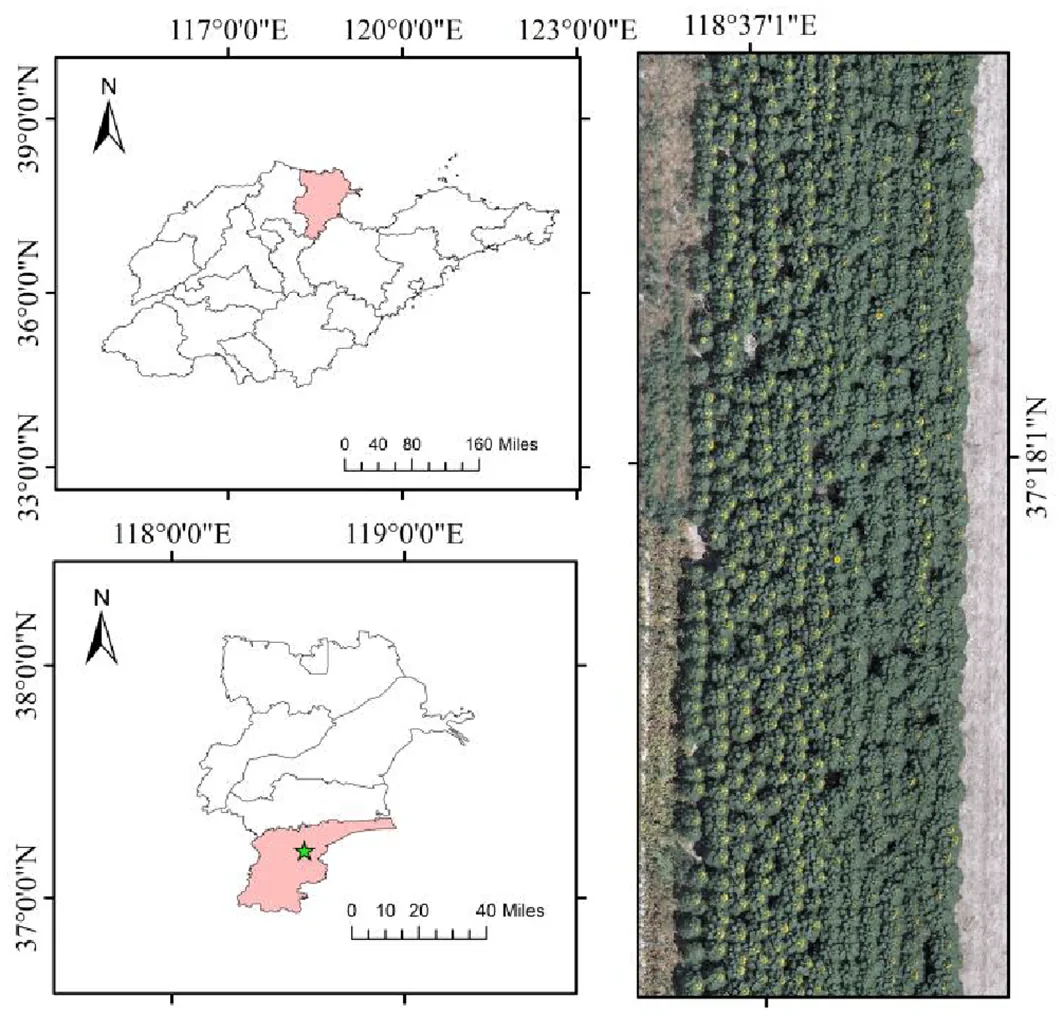
Map of the study area.

The field experiment was conducted at the Modern Agriculture Research Institute of the Yellow River Delta, Guangrao County, Dongying City, Shandong Province, China. The study area is located at 36.4°–37.1°N and 118.2°–119.1°E. The terrain of the experimental area is relatively flat and gently slopes from south to north. The soil is mainly loess, with a loose texture and relatively high fertility. The region has a typical warm temperate monsoon climate, with an annual mean temperature of 13–15 °C and annual precipitation of approximately 800–900 mm. The sunflower planting field was 100 m in length and 10 m in width, covering an area of approximately 1,000 m^2^. The location and field layout of the study area are shown in **Figure 1**.

### 2.2 Data acquisition and preprocessing

**Figure 2:**
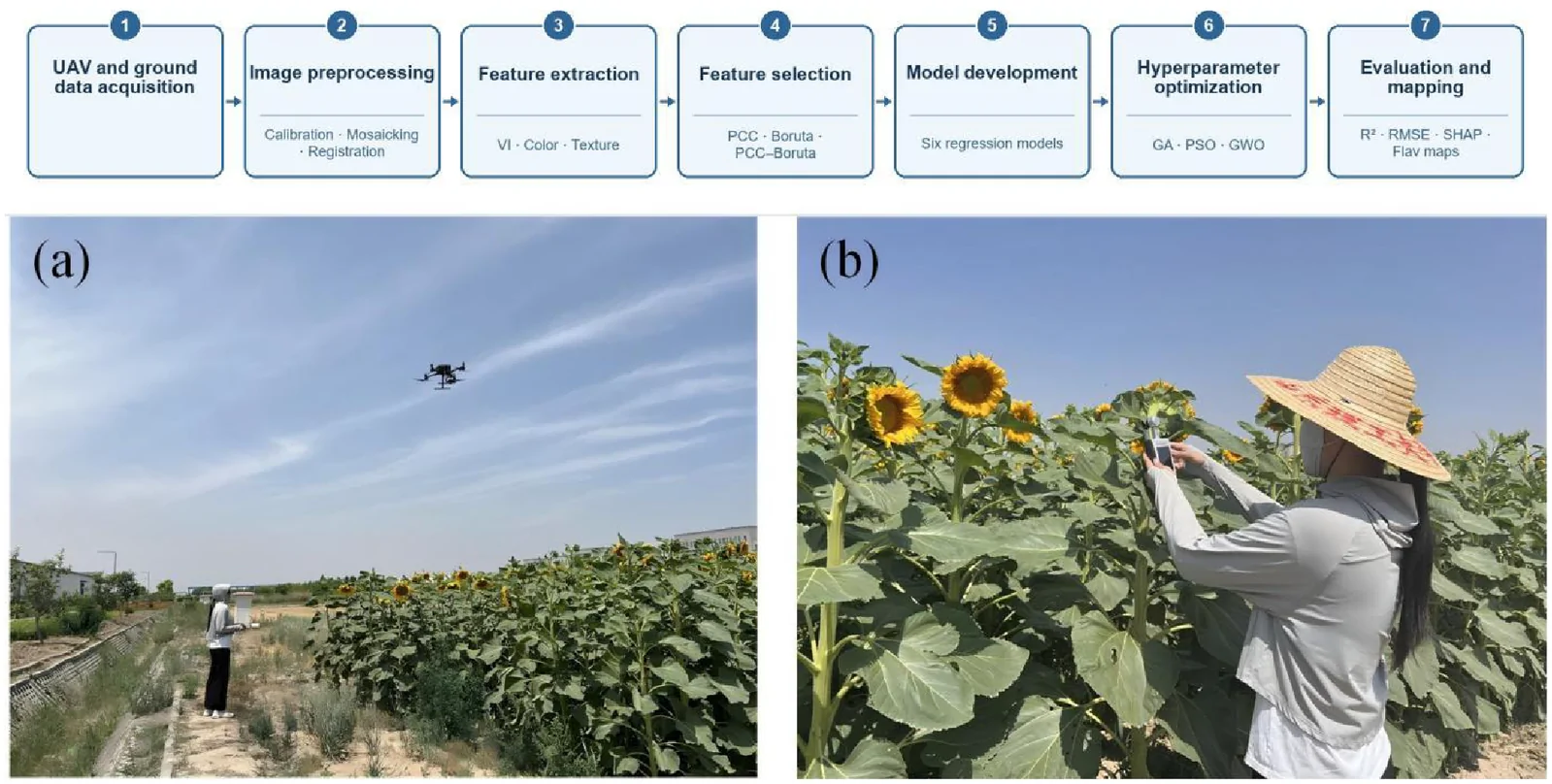
Workflow of UAV-based sunflower leaf Flav estimation. **(a) **UAV remote sensing data collection; **(b)** ground measurement of sunflower leaf Flav.

UAV remote sensing images and ground measurements of sunflower leaf Flav were collected within the same acquisition window at four key growth stages: the seedling, budding, flowering, and maturity stages. The corresponding acquisition dates were 11 April, 22 May, 8 June, and 3 July 2025, respectively. To reduce the influence of illumination variation and environmental disturbance, all UAV flights were conducted between 10:00 and 14:00 under clear weather and low-wind conditions. The overall workflow of UAV-based sunflower leaf Flav estimation, including data acquisition, image preprocessing, feature extraction, feature selection, model development, optimization, and evaluation, is illustrated in **Figure 2**.

#### 2.2.1 UAV image acquisition

Visible-light and multispectral UAV images of the sunflower canopy were acquired using two UAV platforms. A DJI M300 UAV equipped with a Zenmuse P1 visible-light camera (both manufactured by SZ DJI Technology Co., Ltd., Shenzhen, China) was used to acquire high-resolution RGB imagery of the sunflower canopy. The Zenmuse P1 camera has a 45.2 MP CMOS image sensor, providing high spatial resolution for the extraction of canopy color and spatial features.

Multispectral imagery was acquired using a DJI M210 UAV equipped with an MS600 multispectral camera developed by Chang Guang Yuchen Co., Ltd. (Qingdao, China). The MS600 camera contains six spectral channels: blue, green, red, red-edge, and two near-infrared bands, with center wavelengths of 450, 555, 660, 710, 840, and 940 nm, respectively. A camera-mounted irradiance sensor was used to synchronously record incident solar irradiance during each multispectral flight, providing auxiliary information for correcting illumination variations among acquisition periods and flight heights.

At each growth stage, UAV images were acquired at three flight heights of 30 m, 50 m, and 80 m. The flight speed was set to 2.5 m s^−1^, and both the longitudinal and lateral overlaps were set to 85%. At each growth stage, the RGB and multispectral flight missions were conducted sequentially rather than simultaneously. The second flight mission commenced approximately 5 min after completion of the first mission. Both missions were completed within the same short acquisition period under clear and relatively stable illumination conditions to minimize temporal differences between the RGB and multispectral observations.

#### 2.2.2 Radiometric calibration and image preprocessing

After data acquisition, the original images were subjected to quality inspection, and images affected by motion blur, overexposure, or abnormal UAV attitude were removed. For each acquisition date and flight height, corresponding images of a calibrated reflectance reference panel with known band-specific reflectance values were used for multispectral radiometric calibration. The synchronized irradiance measurements were used together with the reference-panel images to account for temporal variations in incident illumination during multispectral image acquisition. Based on the reference-panel images and the known reflectance values of the panel, the raw digital number (DN) values of the multispectral images were converted to reflectance using the manufacturer-provided processing software supplied with the MS600 V2. Radiometric calibration was conducted separately for each acquisition to reduce radiometric differences caused by variations in acquisition date, illumination conditions, exposure, and flight height. Therefore, the subsequent multispectral vegetation indices and related features were calculated from calibrated reflectance images rather than from uncorrected DN images.

Geometric correction and orthomosaic generation were subsequently performed using Pix4Dmapper to produce RGB orthomosaics and multispectral reflectance orthomosaics. To further reduce spatial brightness and illumination differences within the multispectral images, the reflectance image of each band was imported into ENVI 5.3. A region of interest with relatively uniform reflectance was selected as the reference region, and flat-field correction was applied separately to each spectral band.

After flat-field correction, the multispectral bands were accurately co-registered and stacked. The RGB orthomosaic was used as the reference image, whereas the multispectral reflectance orthomosaic was used as the image to be registered. Before registration, both datasets were transformed to the same projected coordinate system. For each RGB–multispectral image pair, image-to-image registration was performed in ENVI 5.3 using approximately 15–20 evenly distributed tie points selected from stable and clearly identifiable ground features, including plot corners, field boundaries, and road intersections. Tie points with large residual errors were iteratively removed, and an affine transformation was applied. The final registration root mean square error (RMSE) was below 0.5 output pixels.

During geometric registration and spatial-resolution standardization, nearest-neighbor resampling was used to avoid interpolation-induced changes in the original pixel values, particularly the multispectral reflectance values. The RGB and multispectral orthomosaics acquired at flight heights of 30 m, 50 m, and 80 m were resampled to a common spatial resolution of 6 cm pixel^−1^. The output images were aligned to the same projected coordinate system, spatial extent, pixel size, and pixel grid.

After registration and resampling, identical georeferenced plot-boundary polygons were applied to the RGB and multispectral orthomosaics at all growth stages and flight heights. This ensured that the RGB color features and multispectral vegetation-index and texture features corresponded to the same physical sampling plots. The extracted features were subsequently matched according to plot ID and fused for plot-level sunflower leaf Flav estimation.

#### 2.2.3 Ground measurement of sunflower leaf Flav

To improve the representativeness of ground measurements, the experimental field was divided into 80 sampling plots, each measuring 2.5 m × 5 m. Sunflower leaf Flav was measured using an MPM-100 plant multispectral pigment measurement device. The instrument estimates Flav based on fluorescence spectroscopy using the fluorescence signal ratio F660 nm/F325 nm.

For each sampled sunflower plant, leaves from different canopy positions were selected to reduce the influence of within-plant heterogeneity. Each leaf was measured at several positions, and five independent readings were collected at each measurement position. The average of the five readings was used as the Flav value of the corresponding measurement point. The final Flav of each sunflower sample was calculated by averaging the measurement results from the selected leaves. This procedure was used to reduce random measurement error and improve the reliability of the ground reference data.

### 2.3 Feature extraction from UAV imagery

To describe the spectral response, color characteristics, and spatial texture structure of the sunflower canopy, three types of features were extracted from the UAV images: vegetation indices, texture features, and color features. In total, 44 remote sensing features were calculated, comprising 22 vegetation indices, 16 texture features, and six color features.

#### 2.3.1 Sunflower canopy segmentation

Before canopy feature extraction, sunflower canopy pixels were separated from soil, non-vegetation shadows, weeds, and other background elements. For each growth stage and flight height, the normalized difference vegetation index (NDVI) was calculated from the radiometrically corrected red and near-infrared bands centered at 660 and 840 nm, respectively. An image-specific threshold was determined using Otsu’s method and applied to generate an initial binary vegetation mask. The initial mask was refined using morphological opening and closing, hole filling, and the removal of small isolated components. This procedure removed bare soil, soil shadows, and other non-vegetation background pixels. Obvious weed patches were further excluded based on their spatial positions relative to the sunflower rows and visual inspection of the corresponding RGB orthomosaics. Shaded sunflower leaf pixels that satisfied the vegetation criterion were retained because they represented part of the actual crop canopy.

Because the RGB and multispectral orthomosaics had been geometrically registered and resampled to the same spatial resolution and pixel grid, the final sunflower canopy mask was applied consistently to both datasets. Background pixels were assigned as NoData and excluded from feature calculation. RGB color features and multispectral vegetation-index and texture features were therefore extracted only from valid sunflower canopy pixels within the same georeferenced plot boundaries.

#### 2.3.2 Vegetation indices

Vegetation indices were calculated from visible-light and multispectral bands to quantify sunflower canopy growth status, chlorophyll-related spectral response, physiological stress, and the influence of soil background. In this study, 22 vegetation indices were selected, comprising visible-light vegetation indices and multispectral vegetation indices. The definitions and calculation formulas of the 22 vegetation indices used in this study are summarized in **Table 1**. The selected indices were the Green–Red Ratio Index (GRRI), Color Index for Vegetation Extraction (CIVE), Excess Red Index (EXR), Excess Green Index (EXG), Green–Blue Difference Index (GBDI), Kawashima Index (IKAW), Modified Green–Red Vegetation Index (MGRVI), Normalized Green–Blue Difference Index (NGBDI), Red–Green Ratio Index (RGRI), Visible Atmospherically Resistant Index (VARI), Green–Red Vegetation Index (GRVI), Red-edge Chlorophyll Index (CIre), Green Chlorophyll Vegetation Index (CIg), Green Normalized Difference Vegetation Index (GNDVI), Generalized Soil Adjusted Vegetation Index (GSAVI), Modified Chlorophyll Absorption Ratio Index (MCARI), Modified Nonlinear Vegetation Index (MNLI), Modified Soil Adjusted Vegetation Index (MSAVI), Normalized Difference Red-edge Index (NDRE), Ratio Vegetation Index (RVI), Transformed Vegetation Index (TVI), and Weighted Difference Vegetation Index (WDVI).

**Table 1:** Vegetation indices used for sunflower leaf Flav estimation.

Vegetation index type	Vegetation index	Calculation formula	References
Visible-light vegetation index	Green-Red Ratio Index (GRRI)	GRRI=G/R	(Elsayed et al., 2021)
	Color Index for Vegetation Extraction (CIVE)	CIVE=0.441R−0.811G+0.385B+18.787	(Ponti, 2013)
	Excess Red Index (EXR)	EXR=1.4R-G	(Meyer and Neto, 2008)
	Excess Green Index (EXG)	EXG=2G-R-B	(Woebbecke et al., 1995)
	Green-Blue Difference Index (GBDI)	GBDI=G−B	(Woebbecke et al., 1995)
	Kawashima Index (IKAW)	IKAW=(R−B)/(R+B)	(Kawashima and Nakatani, 1998)
	Modified Green-Red Vegetation Index (MGRVI)	$\text{MGRVI}=\left({\text{G}}^{2}-{\text{R}}^{2}\right)/\left({\text{G}}^{2}+R^{2}\right)$	(Bendig et al., 2015)
	Normalized Green-Blue Difference Index (NGBDI)	NGBDI=(G−B)/(G+B)	(Verrelst et al., 2008)
	Red-Green Ratio Index (RGRI)	RGRI=R/G	(Gamon and Surfus, 1999)
	Visible Atmospherically Resistant Index (VARI)	VARI=(G−R)/(G+R−B)	(Gitelson et al., 2003)
	Green-Red Vegetation Index (GRVI)	GRVI=(G−R)/(G+R)	(Gitelson et al., 2005)
Multispectral vegetation index	Red-edge Chlorophyll Index (CIre)	CIre=(nir/RE)−1	(Li et al., 2024)
	Green Chlorophyll Vegetation Index (CIg)	CIg=(nir/G)−1	(Gitelson et al., 1996a)
	Green Normalized Difference Vegetation Index (GNDVI)	GNDVI=(nir−G)/(nir+G)	(Gitelson et al., 1996b)
	Generalized Soil Adjusted Vegetation Index (GSAVI)	GSAVI=[(1+L)×(nir−RE)]/(nir+RE+L)]	(Niu et al., 2021)
	Modified Chlorophyll Absorption Ratio Index (MCARI)	MCARI=[RE−R−0.2(RE−G)]×RE/R	(Wu et al., 2008)
	Modified Nonlinear Vegetation Index (MNLI)	$MNLI=1.5\left(nir^{2}-RE\right)/\left(nir^{2}+RE+0.5\right)$	(Gong et al., 2003)
	Modified Soil-Adjusted Vegetation Index (MSAVI)	$\text{MSAVI=}\left[\left(2nir+1\right)-\sqrt{{\left(2nir+1\right)}^{2}-8\left(\text{nir-R}\right)}\right]/2$	(Qi et al., 1994)
	Normalized Difference Red-edge Index (NDRE)	NDRE=(nir−RE)/(nir+RE)	(Gitelson and Merzlyak, 1994)
	Ratio Vegetation Index (RVI)	RVI=nir/R	(Pearson and Miller, 1972)
	Transformed Vegetation Index (TVI)	$TVI=\sqrt{\frac{nir-RE}{nir+RE}+0.5}$	(Perry and Lautenschlager, 1984)
	Weighted Difference Vegetation Index (WDVI)	WDVI=nir−1.5R	(Ehammer et al., 2010)

R, G, and B represent the red, green, and blue bands centered at 660, 555, and 450 nm, respectively; RE represents the red-edge band centered at 710 nm; nir represents the near-infrared band centered at 840 nm; and L is the soil-adjustment factor, which was set to 0.5. The 940 nm band was not used in the vegetation-index calculations.

In the formulas, R, G, and B represent the red, green, and blue bands centered at 660, 555, and 450 nm, respectively; RE represents the red-edge band centered at 710 nm; nir represents the near-infrared band centered at 840 nm; and L represents the soil adjustment factor, which was set to 0.5 in this study. The 840 nm band was used for all vegetation-index calculations requiring an nir band, whereas the 940 nm band was not included in the vegetation-index feature set.

#### 2.3.3 Texture features

Texture features were extracted to characterize the spatial distribution, local heterogeneity, and structural information of the sunflower canopy. The gray-level co-occurrence matrix (GLCM) method was used for texture feature extraction (Yuan et al., 2019; Li et al., 2023). Considering the sensitivity of the red-edge and near-infrared bands to vegetation canopy structure and biochemical variation, GLCM texture features were extracted separately from the 710 nm red-edge band and the 840 nm near-infrared band. The 940 nm band was not used for texture-feature extraction.

The pixel values of each band were quantized into eight gray levels. A 3×3-pixel moving window, corresponding to a ground area of approximately 18×18 cm, was used, and the window was moved across the image with a step size of one pixel. Within each window, the GLCM was constructed using a pixel-pair distance of one pixel and a direction of 45°. Eight GLCM texture metrics were calculated for each band: mean, variance, homogeneity, contrast, dissimilarity, entropy, second moment, and correlation. Consequently, eight texture features were obtained from each band, resulting in a total of 16 texture features. For each sampling plot, the mean value of the valid canopy pixels in each texture-feature image was calculated as the corresponding plot-level texture feature. These texture features supplemented the spectral vegetation indices by providing information on canopy spatial arrangement and image heterogeneity.

#### 2.3.4 Color features

Color features were extracted from RGB imagery using the HSV and LAB color spaces (Wang et al., 2022; Song et al., 2023). The HSV color space describes color according to hue (H), saturation (S), and value (V), which are closely related to human visual perception. The LAB color space includes luminance (L), the green–red component (A), and the blue–yellow component (B), and can represent subtle color changes while reducing the influence of illumination conditions.

Because changes in leaf Flav may be accompanied by variations in canopy color and leaf visual appearance, six color features, including H, S, V, L, A, and B, were extracted from the sunflower canopy images and used as candidate variables for Flav estimation.

### 2.4 Feature selection

After the plot-level training–testing split, PCC analysis and Boruta were independently applied to the training data at each flight height to reduce feature redundancy and identify informative predictors.

Pearson correlation coefficient analysis was used to evaluate the linear relationship between each remote sensing feature and ground-measured Flav (Pearson, 1895; Han et al., 2023). The Pearson correlation coefficient was calculated as follows:


$$r=\frac{\sum_{i=1}^{n}\left(x_{i}-\bar{x}\right)\left(y_{i}-\bar{y}\right)}{\sqrt{\sum_{i=1}^{n}{\left(x_{i}-\bar{x}\right)}^{2}}\sqrt{\sum_{i=1}^{n}{\left(y_{i}-\bar{y}\right)}^{2}}}$$

where *x_i_* represents the value of a remote sensing feature, *y_i_* represents the measured Flav, $\bar{x}$ and $\bar{y}$ are the corresponding mean values, and *n* is the number of samples. Features with higher absolute correlation coefficients were considered more sensitive to variations in sunflower Flav.

The Boruta algorithm was used as a model-based feature selection method (Kursa and Rudnicki, 2010; Kursa et al., 2010). Boruta is based on random forest and evaluates the importance of original features by comparing them with randomly generated shadow features. Features confirmed by Boruta were considered important variables for Flav estimation. Compared with correlation-based feature selection, Boruta can better capture nonlinear relationships and interactions among feature variables.

Feature-number determination and final feature-subset construction were conducted exclusively within the 56-plot training dataset. Grouped five-fold cross-validation was used to determine the number of features retained separately for each flight height, feature category, and feature-selection method. Plot ID was used as the grouping variable so that all four growth-stage observations from the same plot were assigned to the same fold. Within each fold, PCC values and Boruta importance rankings were recalculated using only the internal training plots. The ranked features were then sequentially introduced into each of the six regression models, and predictive performance was evaluated using the corresponding internal validation plots. For each candidate feature number, validation R^2^ was first averaged across the five folds for each regression model and was subsequently averaged across the six models. The feature number corresponding to the highest overall mean validation R^2^ was selected as the retained number for that flight height, feature category, and feature-selection method. Thus, the retained feature number was determined independently for each selection scenario but was kept consistent across the six regression models to ensure a fair model comparison.

After the retained feature numbers had been determined, PCC and Boruta were rerun using the complete 56-plot training dataset to obtain the final feature rankings. For each flight height and feature category, the corresponding top-ranked PCC and Boruta features were retained according to their cross-validation-determined feature numbers. The intersection of the truncated PCC and Boruta subsets was then used to construct the final PCC-Boruta predictor set. The 24-plot testing dataset was not used for feature ranking, feature-number determination, or final feature-subset construction.

### 2.5 Model construction and optimization

#### 2.5.1 Dataset division and model inputs

A total of 320 plot-level observations were obtained from 80 sampling plots across four sunflower growth stages, with one observation collected from each plot at each growth stage. To prevent information leakage caused by repeated observations from the same sampling plot, the dataset was divided at the plot level rather than at the individual-observation level. Specifically, the 80 sampling plots were randomly divided into 56 training plots and 24 testing plots at a ratio of 7:3. All observations from the same plot across the seedling, budding, flowering, and maturity stages were assigned exclusively to either the training set or the testing set. Consequently, the training set contained 224 observations, whereas the testing set contained 96 observations. The same plot-level division was consistently applied to the datasets acquired at flight heights of 30 m, 50 m, and 80 m, ensuring that comparisons among flight heights, feature-selection strategies, feature combinations, and regression models were based on identical training and testing plots. The plot-level division was completed before feature selection and model construction. Pearson correlation coefficient analysis and Boruta feature selection were conducted exclusively using the training dataset at each flight height. The testing dataset was not involved in feature ranking, feature selection, or model fitting. The feature subsets identified from the training dataset were subsequently applied to the corresponding training and testing datasets. Regression models were fitted using the training dataset, whereas the testing dataset was used for comparative performance evaluation.

For each flight height, the UAV-derived features were matched with the corresponding ground-measured Flav values according to plot ID and growth stage. Based on the selected vegetation indices, color features, and texture features, four feature sets were constructed:

- vegetation indices + color features;
- color features + texture features;
- vegetation indices + texture features;
- vegetation indices + color features + texture features.

These feature sets were used to compare the contributions of different feature combinations to sunflower Flav estimation across the three UAV flight heights.

#### 2.5.2 Regression models

Six regression algorithms were used to construct sunflower leaf Flav estimation models: K-nearest neighbors (KNN), random forest (RF), CatBoost, support vector regression (SVR), Light Gradient Boosting Machine (LightGBM), and Transformer regression. All models were implemented in the Jupyter Notebook environment.

KNN was used as a baseline regression model because of its simple structure and suitability for small-sample datasets. RF, CatBoost, and LightGBM were selected as ensemble learning models because they can capture nonlinear relationships and interactions among remote sensing features. SVR was selected because of its strong performance in nonlinear regression under limited sample conditions. Transformer regression was introduced to explore the potential of the self-attention mechanism for modeling complex relationships among multi-source UAV features.

For each flight height and feature set, the six models were trained using the training set and evaluated using the same testing set (Cover and Hart, 1967; Breiman, 2001; Cortes and Vapnik, 1995; Vaswani et al., 2017; Ke et al., 2017; Prokhorenkova et al., 2018). Their performance was compared across flight heights, feature-selection strategies, and feature sets. The regression algorithm with the best overall comparative performance was selected for subsequent hyperparameter optimization.

#### 2.5.3 Optimization algorithms

To further improve the predictive performance of the Transformer model, three established metaheuristic optimization algorithms were compared: the genetic algorithm (GA), particle swarm optimization (PSO), and grey wolf optimizer (GWO). GA searches for optimal solutions through selection, crossover, and mutation. PSO updates candidate solutions according to particle velocity, individual best position, and global best position. GWO simulates the social hierarchy and hunting behavior of grey wolf populations, in which the α, β, and δ wolves guide the position updates of candidate solutions (Mirjalili et al., 2014).

Previous studies have shown that UAV flight-height selection requires a trade-off among image quality, spatial resolution, area coverage, and operational efficiency, and that an intermediate flight height may provide a practical compromise among these factors (Stamenković et al., 2024). Therefore, the 50 m dataset, representing the intermediate condition among the three flight heights evaluated in this study, was selected for subsequent Transformer hyperparameter optimization. Hyperparameter search was conducted exclusively within the plot-level training dataset. Before optimization, the 56 training plots were further divided at the plot level at an approximate ratio of 7:3 into an optimization-training subset containing 39 plots and a validation subset containing 17 plots, using a fixed random seed of 42. All observations from the same plot across the four growth stages were assigned to the same subset, thereby preventing plot overlap between the optimization-training and validation subsets. The feature subset used for hyperparameter optimization had previously been determined using the complete 56-plot training dataset.

The hyperparameters considered in the optimization included the learning rate, batch size, hidden dimension, number of attention heads, number of Transformer encoder layers, and dropout rate. The learning rate was searched within the range of 1×10^−4^ to 5×10^−3^, and the candidate batch sizes were 8, 16, and 32. The candidate hidden dimensions were 16, 32, and 64, whereas the number of attention heads was selected from 1, 2, 4, and 8, subject to the requirement that the hidden dimension be divisible by the number of attention heads. The number of Transformer encoder layers ranged from 1 to 3, and the dropout rate ranged from 0.1 to 0.5. For each candidate hyperparameter combination generated using GA, PSO, or GWO, the Transformer model was trained using the optimization-training subset and evaluated using the validation subset. Validation RMSE was used as the fitness function, and the optimization objective was to minimize this value. For each optimization algorithm, the hyperparameter combination corresponding to the minimum validation RMSE was retained. The resulting GA-, PSO-, and GWO-tuned Transformer models were subsequently retrained using all 56 training plots and evaluated using the 24 testing plots. Hyperparameter selection was based on the validation subset, whereas the testing set was used for comparative evaluation of the three optimized models.

#### 2.5.4 Model evaluation

Model performance was evaluated using the coefficient of determination (R^2^) and the root mean square error (RMSE). R^2^ was used to evaluate the explanatory ability of the model, with higher values indicating better agreement between predicted and measured Flav. RMSE was used to quantify the average prediction error, with lower values indicating higher estimation accuracy (Zhou et al., 2022).

The calculation formulas are as follows:


$$R^{2}=1-\frac{\sum_{i=1}^{n}{\left(y_{i}-{\hat{y}}_{i}\right)}^{2}}{\sum_{i=1}^{n}{\left(y_{i}-\bar{y}\right)}^{2}}$$

$$RMSE=\sqrt{\frac{1}{n}\overset{n}{\underset{i=1}{\sum }}{\left(y_{i}-{\hat{y}}_{i}\right)}^{2}}$$

where *y_i_* is the measured Flav, ${\hat{y}}_{i}$ is the predicted Flav, $\bar{y}$ is the mean measured Flav, and n is the number of samples.

All regression models, feature-selection strategies, feature sets, and flight-height datasets were comparatively evaluated using the same testing set, comprising 96 observations from 24 plots. R^2^ and RMSE were calculated from identical pairs of measured and predicted Flav values. RMSE was used as the primary model-comparison criterion, with R^2^ serving as a complementary metric, whereas validation RMSE was used exclusively for hyperparameter selection within the training dataset. Accordingly, the flight height with the lowest testing-set RMSE, supported by its corresponding R^2^, was considered to provide the highest observed predictive performance among the evaluated unoptimized models. The 50 m dataset, representing the intermediate flight height among the three evaluated levels, was subsequently used for hyperparameter optimization.

#### 2.5.5 Model interpretation and visualization

To improve model interpretability, SHapley Additive exPlanations (SHAP) analysis was used to quantify the contribution of each input feature to model predictions. SHAP values are derived from Shapley values in cooperative game theory and represent the marginal contribution of each feature to the model output. In this study, SHAP analysis was performed for the optimal PCC-Boruta-Transformer model at each flight height to identify the key UAV-derived features affecting sunflower Flav estimation and to examine how feature contributions varied among flight heights.

After the predictive performance of the selected GWO-tuned Transformer model at 50 m had been evaluated using the 24 testing plots, the model was applied to the UAV-derived features of all 80 sampling plots at each growth stage. The resulting plot-level estimates were linked to the corresponding georeferenced plot polygons to generate spatial distribution maps of sunflower leaf Flav. The estimates for the 56 training plots were in-sample estimates, whereas those for the 24 testing plots were out-of-sample predictions. Therefore, the spatial maps were used solely to visualize within-field variation and were not used for additional model evaluation.

## 3 Results

### 3.1 Variation in measured sunflower leaf Flav

The descriptive statistics of measured sunflower leaf Flav at different growth stages are presented in **Table 2**. The measured Flav values showed descriptive variation across the four growth stages. The mean Flav value increased from the seedling stage to the flowering stage and then decreased at the maturity stage. Specifically, the mean Flav values were 1.31, 1.42, 1.54, and 1.18 at the seedling, budding, flowering, and maturity stages, respectively. The highest value was observed at the flowering stage, reaching 2.05, whereas the lowest value occurred at the maturity stage, with a value of 0.68.

**Table 2:** Descriptive statistics of measured sunflower leaf Flav at different growth stages.

Growth stages	Sample size	Maximum value	Minimum value	Average value	Standard deviation	Variance	Coefficient of variation
Seedling stage	80	1.53	0.99	1.31	0.12	0.01	0.09
Budding stage	80	1.76	1.02	1.42	0.10	0.01	0.07
Flowering stage	80	2.05	0.92	1.54	0.23	0.05	0.15
Maturity stage	80	1.86	0.68	1.18	0.29	0.09	0.25

n = 80 plot-level observations at each growth stage. Flav denotes leaf flavonol content measured using an MPM-100 plant multispectral pigment meter.

The coefficients of variation also showed descriptive differences among growth stages. The coefficient of variation was relatively low at the seedling and budding stages, with values of 0.09 and 0.07, respectively, indicating relatively stable Flav levels during early growth. The coefficient of variation increased to 0.15 at the flowering stage and reached the highest value of 0.25 at the maturity stage, suggesting greater sample heterogeneity during the later growth period.

### 3.2 Feature selection results

#### 3.2.1 Pearson correlation-based feature selection

**Figure 3:**
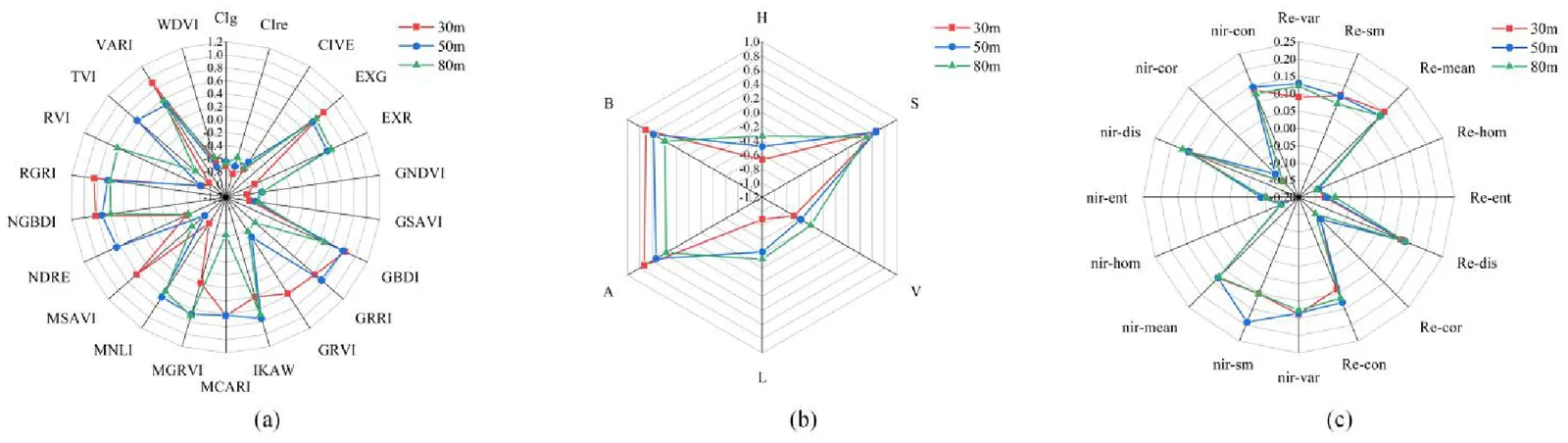
Pearson correlation coefficients between UAV-derived features and sunflower leaf Flav at different flight heights. **(a)** Vegetation indices; **(b)** color features; and **(c)** texture features. All coefficients were calculated using the complete 56-plot training dataset.

The PCC values shown in **Figure 3** were calculated using the complete 56-plot training dataset after the plot-level training–testing split. Vegetation indices generally showed stronger correlations with Flav than color and texture features. At a flight height of 30 m, VARI, GNDVI, TVI, and RGRI showed strong correlations with Flav, with correlation coefficients of 0.892, -0.868, -0.856, and 0.852, respectively. At 50 m, GBDI and MSAVI showed relatively high correlations with Flav, with correlation coefficients of 0.786 and -0.770, respectively. At 80 m, MGRVI and IKAW were the most correlated vegetation indices, with correlation coefficients of 0.706 and 0.687, respectively.

Compared with vegetation indices, texture features showed weaker correlations with Flav. Among them, nir-dis showed relatively stable sensitivity across different flight heights. At 30 m, nir-dis, Re-mean, and nir-hom showed relatively high correlations with Flav. At 50 m, nir-hom, nir-dis, and nir-con showed stronger correlations. At 80 m, nir-dis, Re-hom, and nir-hom were more closely related to Flav. Color features showed moderate correlations with Flav. The S feature showed a relatively high correlation at 50 m, with a correlation coefficient of 0.753, whereas other color features showed weaker correlations.

#### 3.2.2 Boruta-based feature selection

The Boruta feature-importance ranking shown in **Table 3** was obtained using the complete 56-plot training dataset. **Table 3** lists all features confirmed by Boruta before truncation according to the cross-validation-determined feature number. Therefore, the number of features listed in **Table 3** is larger than the number of top-ranked Boruta features subsequently retained for constructing the final feature subsets. The Boruta results indicated that the importance of vegetation indices, color features, and texture features varied with flight height. At 30 m, four vegetation indices and six texture features were excluded. At 50 m, three vegetation indices and seven texture features were excluded. At 80 m, two vegetation indices and six texture features were excluded.

**Table 3:** Feature importance ranking obtained using the Boruta algorithm.

Ranking	Vegetation index			Color feature			Texture feature		
	30 m	50 m	80 m	30 m	50 m	80 m	30 m	50 m	80 m
1	CIVE	MCARI	RVI	B	S	S	nir-hom	RE-var	RE-con
2	GNDVI	CIre	CIg	S	B	B	nir-mean	nir-var	nir-var
3	MCARI	MNLI	EXG	A	H	V	RE-con	RE-dis	RE-dis
4	CIre	GRRI	GBDI	V	V	A	RE-dis	RE-hom	nir-dis
5	GSAVI	IKAW	GRRI	H	A	L	RE-hom	RE-con	RE-var
6	VARI	WDVI	GSAVI	/	L	H	RE-var	nir-dis	RE-hom
7	TVI	GBDI	EXR	/	/	/	nir-con	nir-hom	nir-mean
8	EXR	RVI	GNDVI	/	/	/	nir-dis	nir-con	RE-mean
9	GBDI	MGRVI	CIVE	/	/	/	RE-mean	nir-mean	nir-con
10	RVI	NGBDI	IKAW	/	/	/	nir-var	/	nir-hom
11	NGBDI	MSAVI	MSAVI	/	/	/	/	/	/
12	RGRI	GSAVI	MNLI	/	/	/	/	/	/
13	CIg	RGRI	MCARI	/	/	/	/	/	/
14	MGRVI	CIg	CIre	/	/	/	/	/	/
15	WDVI	CIVE	NGBDI	/	/	/	/	/	/
16	EXG	GRVI	TVI	/	/	/	/	/	/
17	GRRI	NDRE	MGRVI	/	/	/	/	/	/
18	NDRE	VARI	NDRE	/	/	/	/	/	/
19	/	EXR	VARI	/	/	/	/	/	/
20	/	/	RGRI	/	/	/	/	/	/

RE and nir denote the red-edge and near-infrared bands, respectively. Mean, var, hom, con, dis, ent, sm, and cor denote mean, variance, homogeneity, contrast, dissimilarity, entropy, second moment, and correlation, respectively. H, S, and V are HSV color features, whereas L, A, and B are LAB color features. “/” indicates that no feature occurred at the corresponding rank. The table presents all features confirmed and ranked by Boruta using the complete 56-plot training dataset before feature-number truncation. Only the top-ranked features corresponding to the cross-validation-determined feature numbers were retained to construct the Boruta subsets shown in **Figure 7**.

For vegetation indices, CIVE, GNDVI, MCARI, and CIre ranked highly at 30 m; MCARI, CIre, MNLI, and GRRI ranked highly at 50 m; and RVI, CIg, EXG, and GBDI ranked highly at 80 m. For color features, B, S, and A showed relatively high importance at 30 m, whereas S, B, and H ranked highly at 50 m, and S, B, and V ranked highly at 80 m. For texture features, important variables were mainly derived from the red-edge and near-infrared bands, indicating that these bands provided useful spatial information for Flav estimation.

### 3.3 Feature-iteration and common-feature selection results

#### 3.3.1 Feature-iteration results

**Figure 4:**
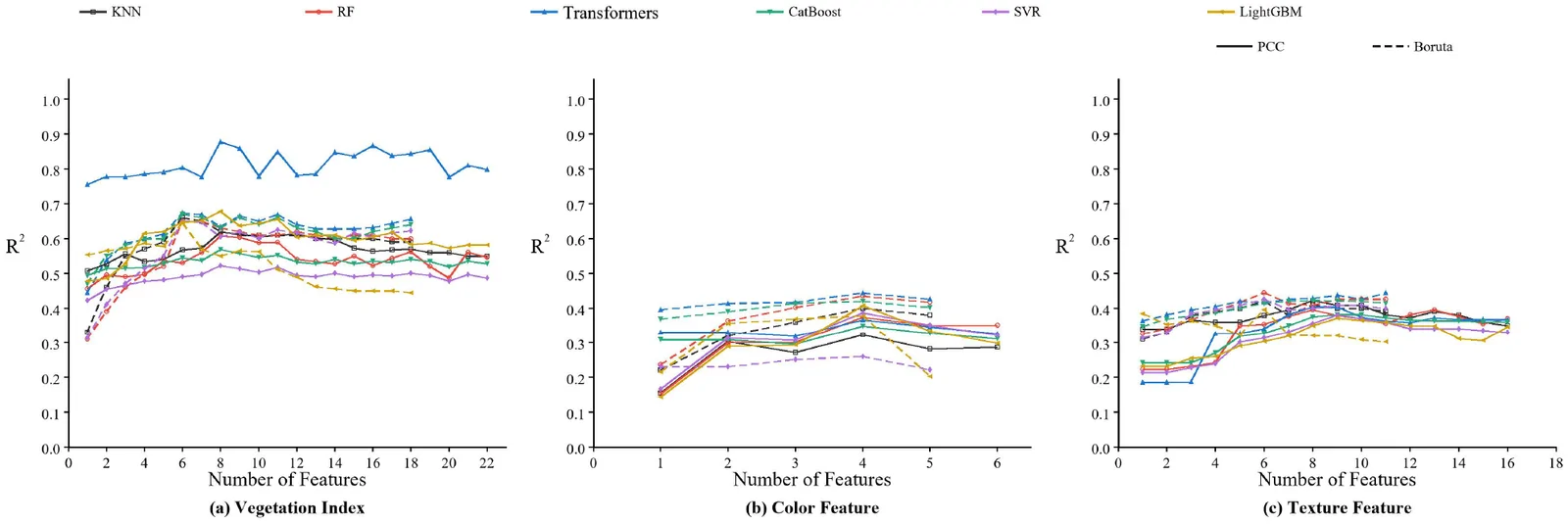
Feature-iteration results at a flight height of 30 m. Curves represent the mean validation R^2^ values obtained from grouped five-fold cross-validation within the training dataset. Solid and dashed lines indicate PCC- and Boruta-based feature selection, respectively. Retained feature numbers were determined as described in Section 2.4. The same conventions apply to **Figures 5** and **6**.

**Figure 5:**
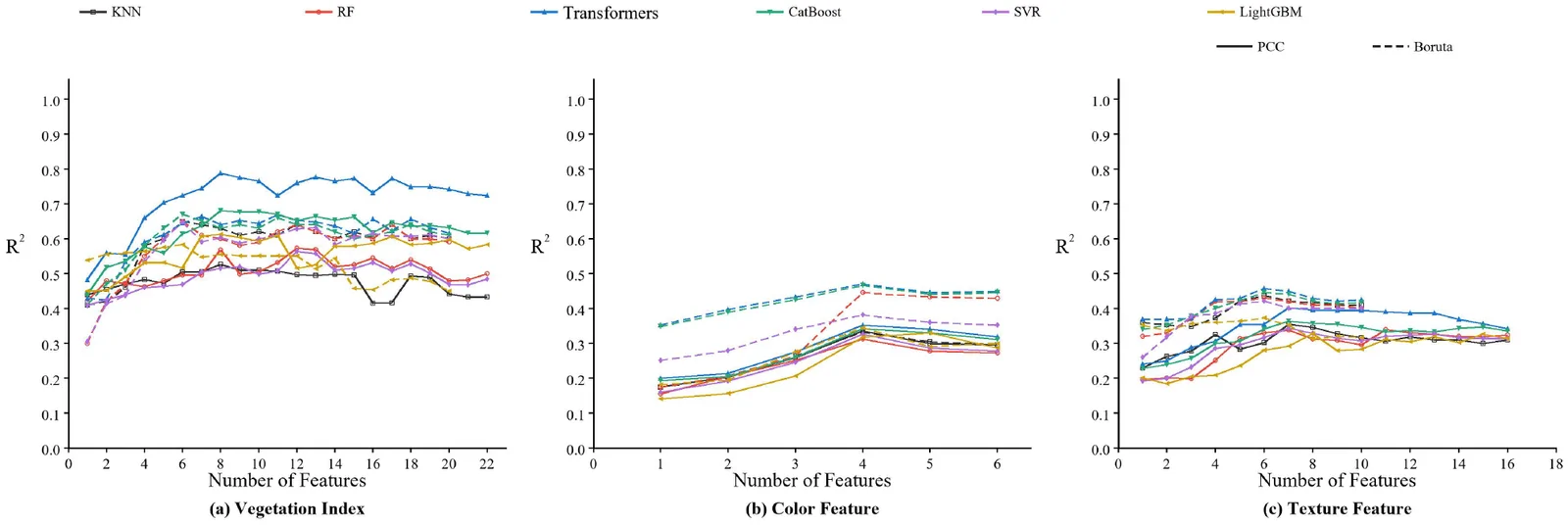
Feature-iteration results at a flight height of 50 m.

**Figure 6:**
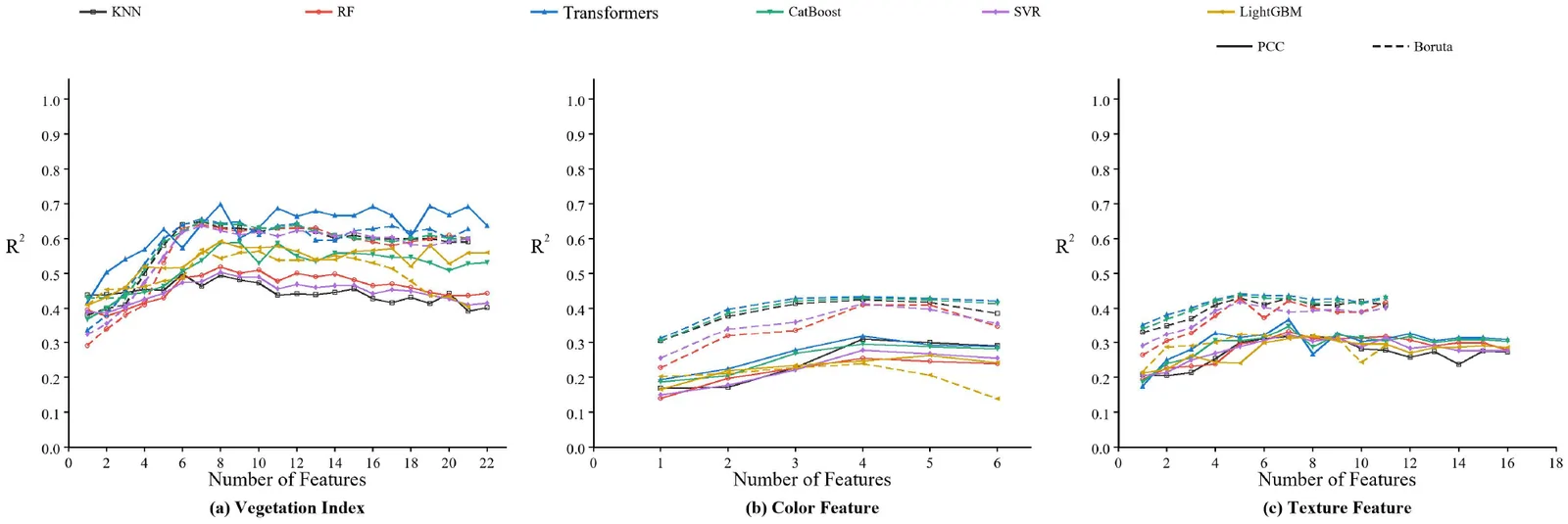
Feature-iteration results at a flight height of 80 m.

**Figures 4**–**6** present the grouped five-fold cross-validation results of PCC- and Boruta-based feature iteration at flight heights of 30 m, 50 m, and 80 m, respectively. Vegetation indices generally produced higher validation R^2^ values than color or texture features, whereas the contributions of color and texture features varied among regression models and flight heights. The retained feature number for each flight height, feature category, and feature-selection method was determined using the overall mean validation R^2^ described in Section 2.4.

#### 3.3.2 Common features selected by PCC and Boruta

**Figure 7:**
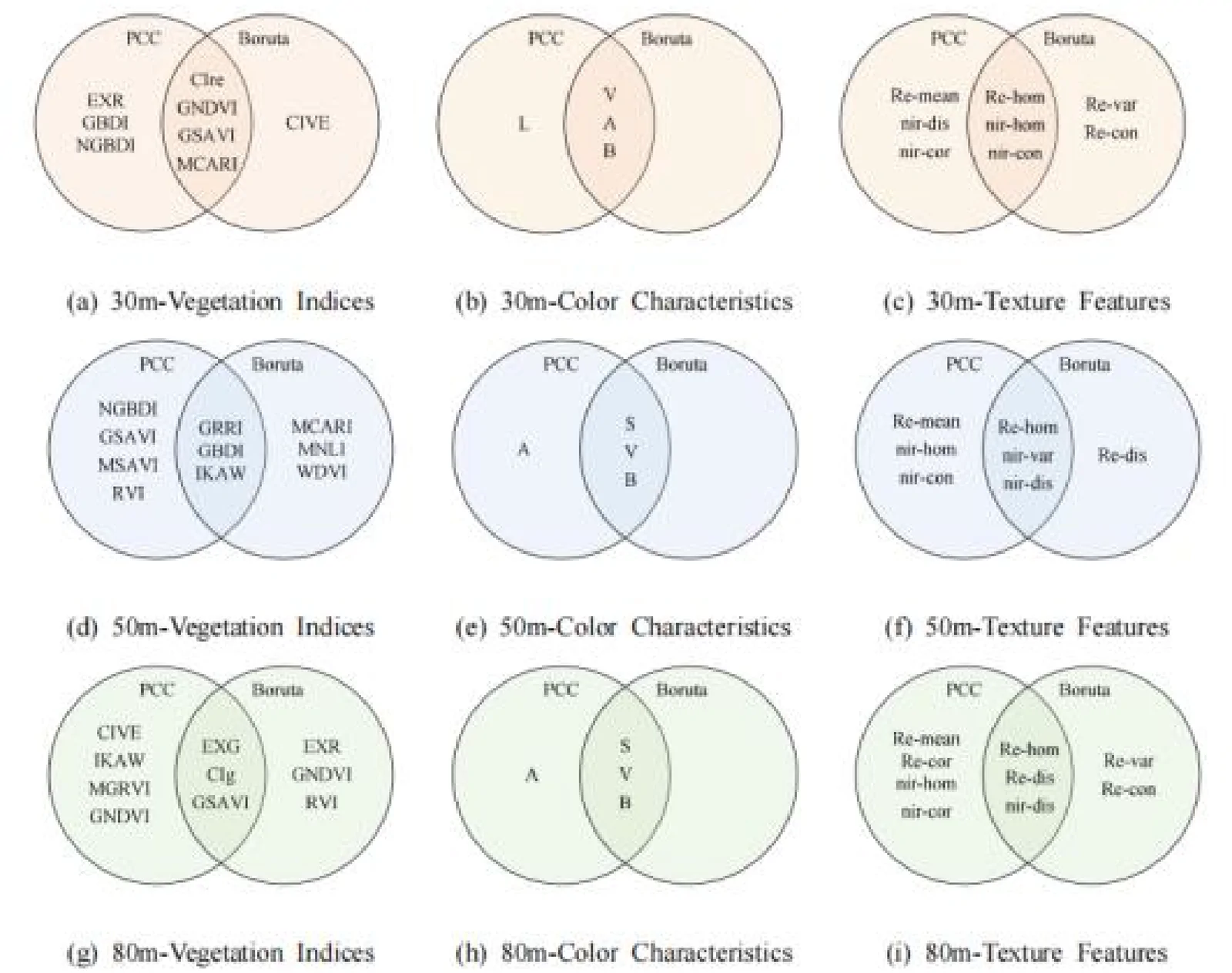
Retained PCC and Boruta feature subsets and their intersections at different flight heights. The retained feature numbers were determined using grouped five-fold cross-validation within the training dataset. PCC and Boruta were then rerun using the complete 56-plot training dataset to identify the corresponding top-ranked features. The left, overlapping, and right regions represent PCC-only, common PCC-Boruta, and Boruta-only features, respectively. The overlapping features constituted the final PCC-Boruta predictor sets.

After the retained feature numbers had been determined using grouped five-fold cross-validation, PCC and Boruta were rerun using the complete 56-plot training dataset. For each flight height and feature category, the corresponding top-ranked features were retained, and the intersection of the truncated PCC and Boruta subsets was used to construct the final PCC-Boruta predictor set shown in **Figure 7**. **Table 3** presents the complete Boruta ranking before feature-number truncation.

The resulting feature intersections contained four vegetation indices, three color features, and three texture features at 30 m. At both 50 and 80 m, three features from each category were retained, resulting in 10, 9, and 9 final predictors at 30 m, 50 m, and 80 m, respectively.

### 3.4 Performance of sunflower Flav estimation models

Four fused feature sets were constructed from the selected vegetation indices, color features, and texture features, as summarized in **Table 4**. Feature sets A, B, and C combined two feature categories, whereas feature set D integrated all three categories. Each feature set was evaluated using KNN, RF, Transformer, CatBoost, SVR, and LightGBM at the three flight heights.

**Table 4:** Definitions of feature sets used for model construction.

Number	Dataset name	Feature name
1	Dataset A	vegetation indices + color features
2	Dataset B	color features + texture features
3	Dataset C	vegetation indices + texture features
4	Dataset D	vegetation indices + color features + texture features

#### 3.4.1 Comparative model performance across flight heights

**Tables 5**–**7** summarize the testing-set performance of the evaluated models at flight heights of 30 m, 50 m, and 80 m, respectively. The corresponding training-set results are provided in **Supplementary Tables 1–S3**. The effect of the PCC-Boruta strategy varied among regression algorithms and feature sets, and PCC or Boruta alone achieved equal or higher predictive performance in some configurations. Nevertheless, the highest observed testing-set performance at each flight height was obtained using the Transformer model, the fully fused feature set D, and the combined PCC-Boruta strategy. The corresponding testing-set R^2^ values were 0.932, 0.911, and 0.909 at 30 m, 50 m, and 80 m, respectively, with RMSE values of 0.060, 0.069, and 0.070. At 80 m, the PCC-Boruta configuration showed only a marginal increase in R^2^ relative to Boruta alone, while the RMSE remained unchanged.

**Table 5:** Testing performance of sunflower leaf Flav estimation models at a flight height of 30 m.

Algorithm	Feature set	PCC		Boruta		PCC-Boruta	
		R^2^	RMSE	R^2^	RMSE	R^2^	RMSE
KNN	A	0.653	0.136	0.655	0.135	0.687	0.129
	B	0.427	0.174	0.431	0.174	0.482	0.166
	C	0.651	0.136	0.668	0.133	0.679	0.131
	D	0.661	0.134	0.685	0.129	0.691	0.128
RF	A	0.633	0.140	0.680	0.130	0.694	0.128
	B	0.428	0.174	0.464	0.169	0.472	0.168
	C	0.635	0.139	0.677	0.131	0.715	0.123
	D	0.677	0.131	0.726	0.121	0.729	0.120
Transformer	A	0.897	0.074	0.798	0.104	0.901	0.073
	B	0.434	0.173	0.473	0.167	0.474	0.167
	C	0.885	0.078	0.790	0.106	0.905	0.071
	**D**	**0.913**	**0.068**	**0.921**	**0.065**	**0.932**	**0.060**
CatBoost	A	0.701	0.126	0.701	0.126	0.760	0.113
	B	0.535	0.157	0.541	0.156	0.602	0.145
	C	0.711	0.124	0.722	0.122	0.732	0.119
	D	0.759	0.113	0.776	0.109	0.858	0.087
SVR	A	0.710	0.124	0.653	0.136	0.628	0.141
	B	0.451	0.171	0.436	0.173	0.540	0.156
	C	0.688	0.129	0.650	0.136	0.667	0.133
	D	0.723	0.121	0.658	0.135	0.690	0.128
LightGBM	A	0.655	0.135	0.685	0.129	0.742	0.117
	B	0.651	0.136	0.705	0.125	0.752	0.115
	C	0.698	0.127	0.680	0.130	0.711	0.124
	D	0.750	0.115	0.762	0.112	0.780	0.108

PCC denotes feature selection based on Pearson correlation coefficients, whereas PCC-Boruta denotes the intersection of the feature subsets selected by PCC and Boruta. All performance metrics were calculated using the same 96 observations from 24 testing plots. Flav is dimensionless, and RMSE is reported on the same dimensionless scale. The row containing the best overall testing-set configuration is shown in bold. The same abbreviations and reporting conventions apply to **Tables 6** and **7**.

**Table 6:** Testing performance of sunflower leaf Flav estimation models at a flight height of 50 m.

Algorithm	Feature set	PCC		Boruta		PCC-Boruta	
		R^2^	RMSE	R^2^	RMSE	R^2^	RMSE
KNN	A	0.630	0.140	0.662	0.134	0.682	0.130
	B	0.334	0.188	0.466	0.168	0.475	0.167
	C	0.599	0.146	0.667	0.133	0.698	0.127
	D	0.657	0.135	0.703	0.126	0.705	0.125
RF	A	0.627	0.141	0.674	0.132	0.685	0.129
	B	0.380	0.182	0.457	0.170	0.466	0.168
	C	0.578	0.150	0.685	0.129	0.694	0.128
	D	0.634	0.139	0.703	0.126	0.711	0.124
Transformer	A	0.793	0.105	0.736	0.118	0.891	0.076
	B	0.365	0.184	0.489	0.165	0.527	0.159
	C	0.859	0.087	0.745	0.116	0.897	0.074
	**D**	**0.877**	**0.081**	**0.871**	**0.083**	**0.911**	**0.069**
CatBoost	A	0.737	0.118	0.763	0.112	0.787	0.106
	B	0.628	0.141	0.647	0.137	0.658	0.135
	C	0.745	0.116	0.771	0.110	0.762	0.112
	D	0.786	0.107	0.808	0.101	0.825	0.096
SVR	A	0.610	0.144	0.602	0.145	0.626	0.141
	B	0.346	0.186	0.537	0.157	0.475	0.167
	C	0.659	0.135	0.608	0.144	0.668	0.133
	D	0.691	0.128	0.615	0.143	0.701	0.126
LightGBM	A	0.630	0.140	0.670	0.132	0.680	0.130
	B	0.534	0.157	0.448	0.171	0.558	0.153
	C	0.650	0.136	0.702	0.126	0.724	0.121
	D	0.680	0.130	0.730	0.120	0.755	0.114

The row containing the best overall testing-set configuration is shown in bold.

**Table 7:** Testing performance of sunflower leaf Flav estimation models at a flight height of 80 m.

Algorithm	Feature set	PCC		Boruta		PCC-Boruta	
		R^2^	RMSE	R^2^	RMSE	R^2^	RMSE
KNN	A	0.533	0.158	0.652	0.136	0.661	0.134
	B	0.302	0.193	0.435	0.173	0.447	0.171
	C	0.566	0.152	0.674	0.132	0.687	0.129
	D	0.608	0.144	0.676	0.131	0.688	0.129
RF	A	0.597	0.146	0.657	0.135	0.662	0.134
	B	0.362	0.184	0.429	0.174	0.456	0.170
	C	0.552	0.154	0.666	0.133	0.679	0.131
	D	0.625	0.141	0.677	0.131	0.698	0.127
Transformer	A	0.827	0.096	0.836	0.093	0.885	0.078
	B	0.385	0.181	0.472	0.168	0.485	0.165
	C	0.817	0.099	0.858	0.087	0.875	0.081
	**D**	**0.864**	**0.085**	**0.907**	**0.070**	**0.909**	**0.070**
CatBoost	A	0.685	0.129	0.736	0.118	0.762	0.112
	B	0.595	0.147	0.591	0.147	0.580	0.149
	C	0.710	0.124	0.730	0.120	0.730	0.120
	D	0.780	0.108	0.800	0.103	0.820	0.098
SVR	A	0.608	0.144	0.600	0.146	0.667	0.133
	B	0.340	0.187	0.337	0.188	0.458	0.170
	C	0.630	0.140	0.597	0.146	0.660	0.134
	D	0.657	0.135	0.611	0.144	0.686	0.129
LightGBM	A	0.640	0.138	0.670	0.132	0.680	0.130
	B	0.420	0.176	0.480	0.166	0.545	0.155
	C	0.660	0.134	0.690	0.128	0.680	0.130
	D	0.708	0.125	0.740	0.118	0.750	0.115

The row containing the best overall testing-set configuration is shown in bold.

**Figure 8:**
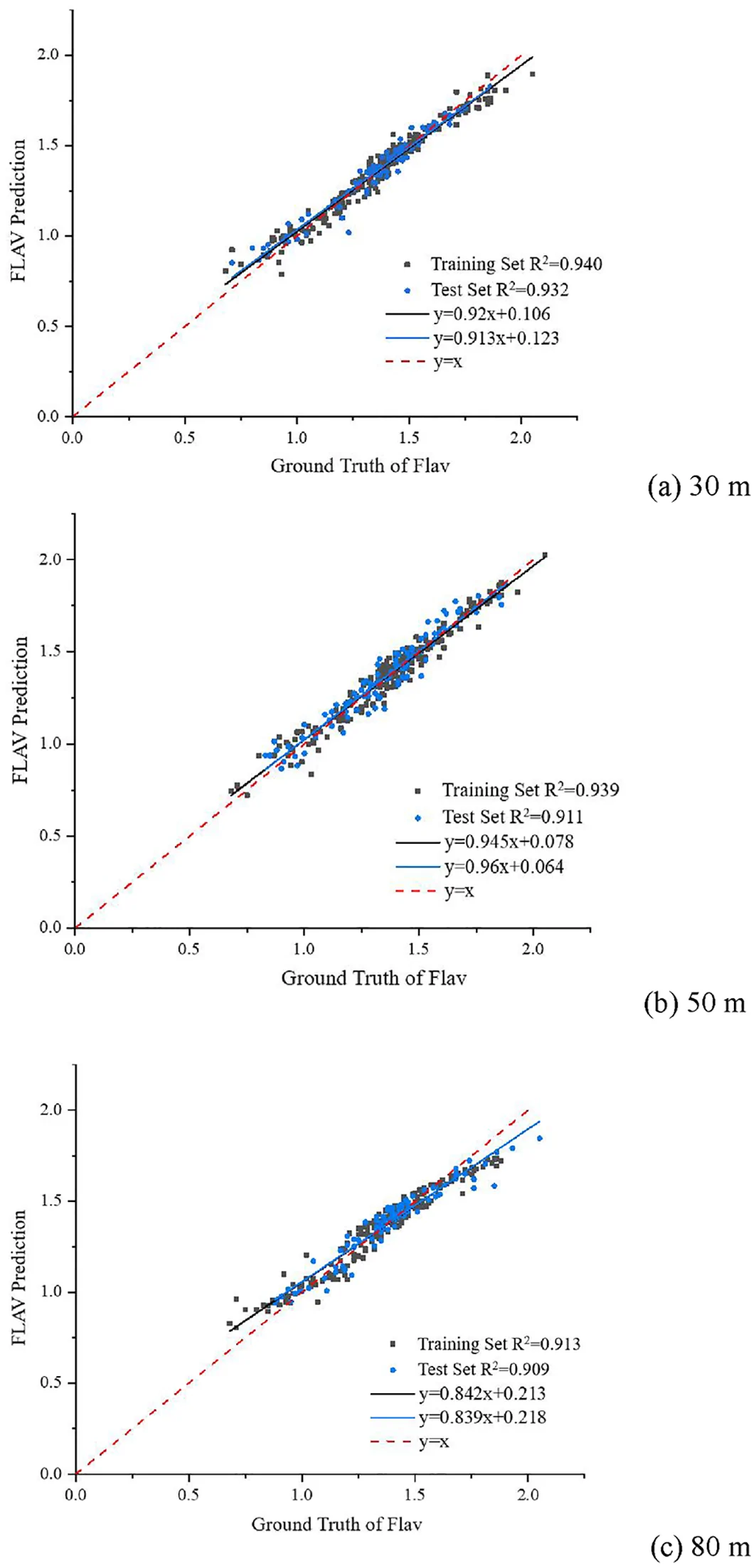
Comparison of measured and predicted sunflower leaf Flav at different flight heights. **(a)** 30 m; **(b)** 50 m; and **(c)** 80 m. The scatter plots show the measured and predicted Flav values for the training and testing sets.

**Figure 8** compares the selected PCC-Boruta-Transformer models across the three flight heights. Among the unoptimized models, the 30 m model achieved the highest observed testing-set performance. Based on the flight-height selection rationale described in Section 2.5.3, the 50 m dataset was subsequently used for hyperparameter optimization.

#### 3.4.2 Model interpretability based on SHAP values

**Figure 9:**
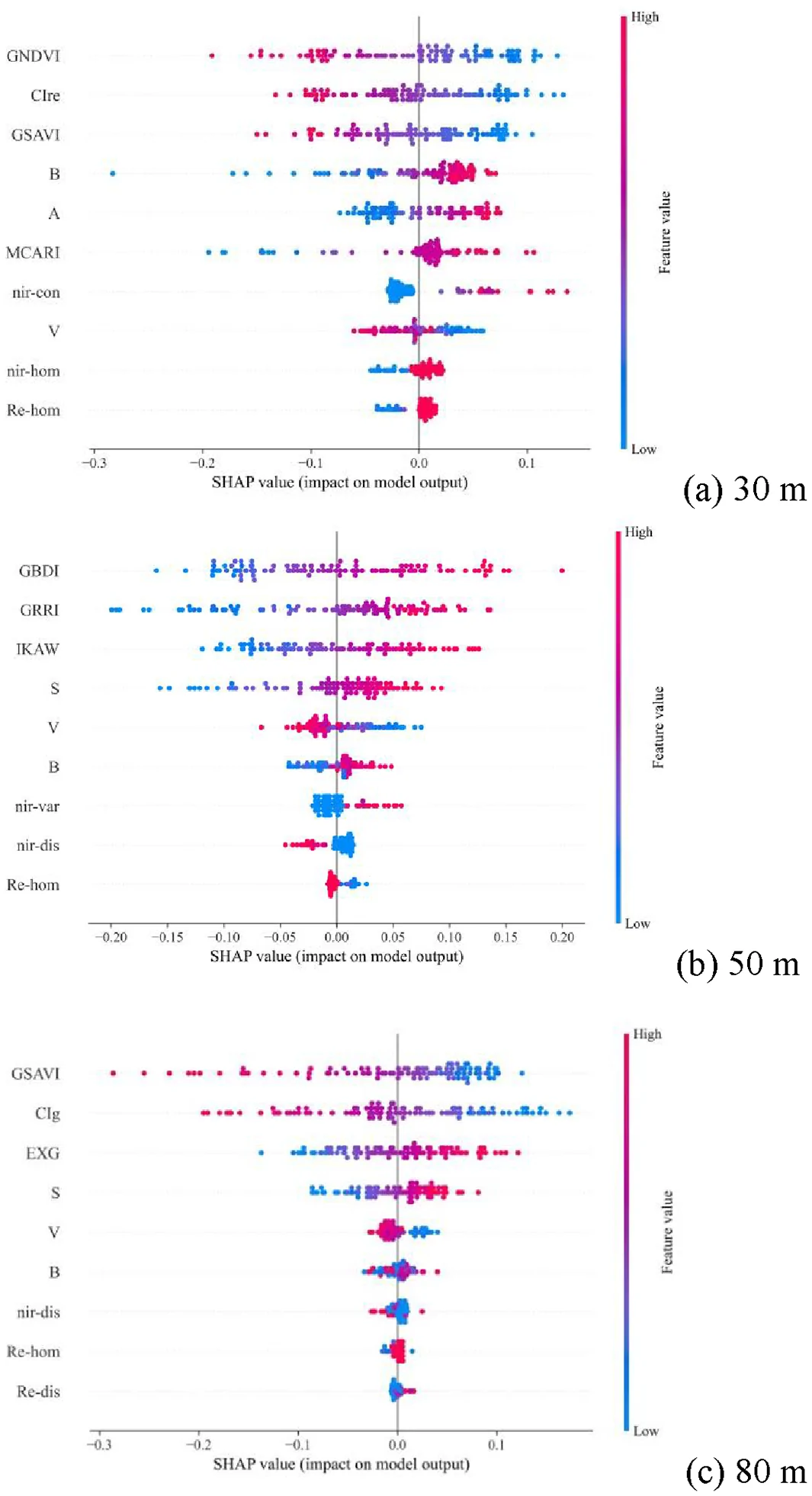
SHAP analysis of the selected sunflower leaf Flav estimation models at different flight heights. **(a)** 30 m; **(b)** 50 m; and **(c)** 80 m. SHAP values quantify the contribution of each UAV-derived feature to model predictions.

The SHAP analysis of the selected models at the three flight heights is shown in **Figure 9**. The dominant predictors varied with flight height: GNDVI, CIre, GSAVI, B, A, and MSAVI contributed strongly at 30 m; GBDI, GRRI, IKAW, S, and V were influential at 50 m; and GSAVI, CIg, EXG, and S showed higher contributions at 80 m. Vegetation indices remained the main predictors, while the occurrence of S, V, B, and A among the important variables indicated that color features provided complementary information. These results indicate that feature contributions were height-dependent and support the use of height-specific feature selection.

### 3.5 Optimization of the transformer model at 50 m

The optimization results of the Transformer model at the intermediate flight height of 50 m are presented in **Table 8**. GA, PSO, and GWO were implemented using the same optimization-training and validation subsets, hyperparameter search space, population size, iteration budget, and model initialization settings. Validation RMSE was used as the fitness function for hyperparameter search. After retraining using all 56 training plots, the GA-, PSO-, and GWO-tuned Transformer models achieved testing-set R^2^ values of 0.917, 0.912, and 0.947, with corresponding RMSE values of 0.066, 0.068, and 0.053, respectively. Among the three evaluated configurations, the GWO-tuned Transformer achieved the highest observed testing-set R^2^ and the lowest testing-set RMSE. Compared with the base Transformer model at 50 m, GWO tuning increased the testing-set R^2^ from 0.911 to 0.947 and reduced the RMSE from 0.069 to 0.053.

**Table 8:** Comparative performance of the optimized transformer models at a flight height of 50 m.

Optimized model	Training set		Testing set	
	R^2^	RMSE	R^2^	RMSE
GA-Transformer	0.942	0.059	0.917	0.066
PSO-Transformer	0.950	0.055	0.912	0.068
**GWO-Transformer**	**0.955**	**0.052**	**0.947**	**0.053**

GA, PSO, and GWO denote genetic algorithm, particle swarm optimization, and grey wolf optimizer, respectively. Validation RMSE served as the fitness criterion during hyperparameter search. After hyperparameter selection, each optimized model was retrained using all 56 training plots and evaluated on the 24 testing plots. The row containing the best overall testing-set configuration is shown in bold.

### 3.6 Spatial distribution of sunflower Flav

**Figure 10:**
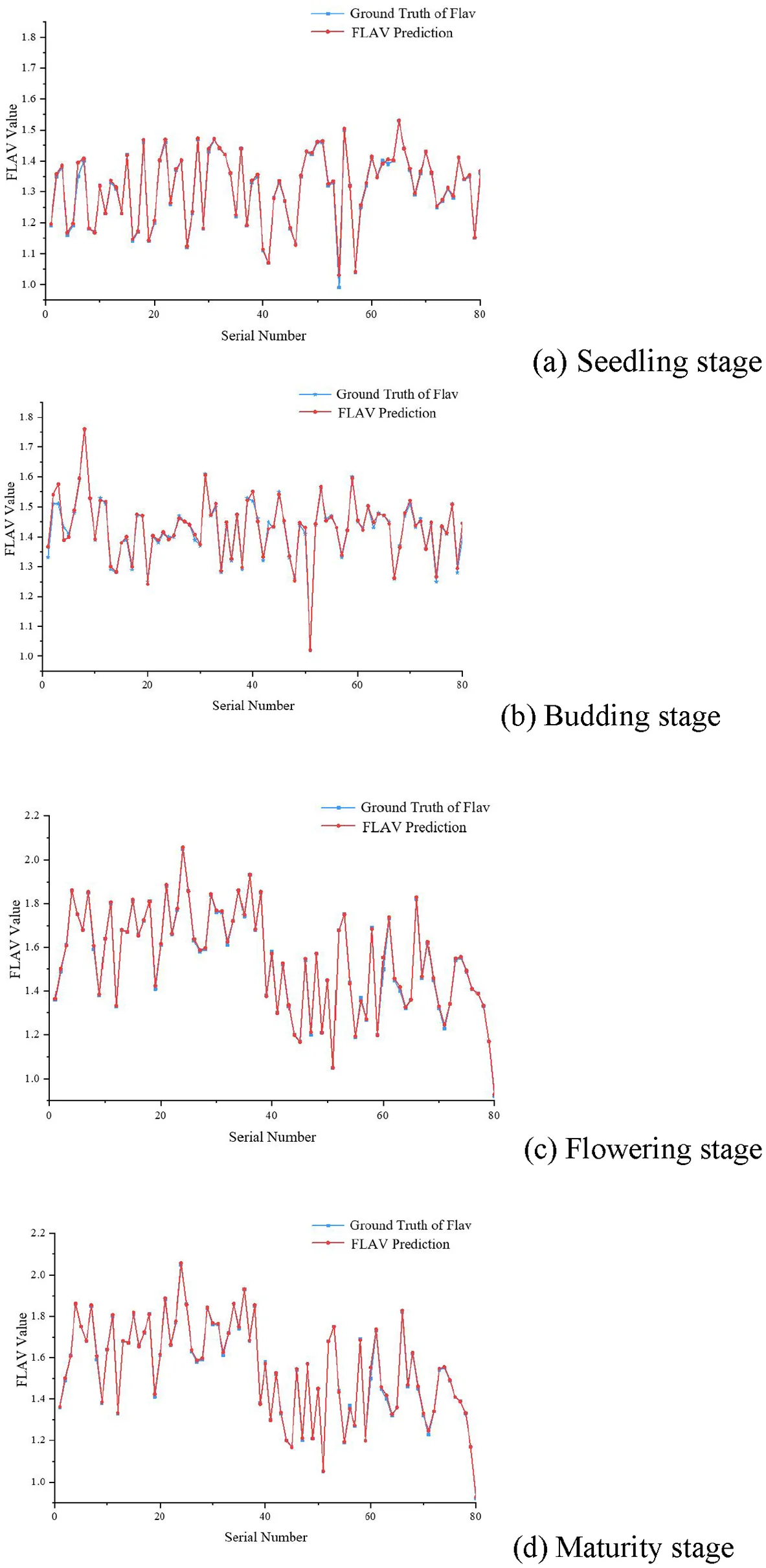
Measured and model-estimated sunflower leaf Flav values across 80 sampling plots at different growth stages. **(a)** Seedling stage; **(b)** budding stage; **(c)** flowering stage; and **(d)** maturity stage. Estimates were generated using the selected GWO-tuned Transformer model with the 50 m dataset and comprised in-sample estimates for the 56 training plots and out-of-sample predictions for the 24 testing plots.

**Figure 11:**
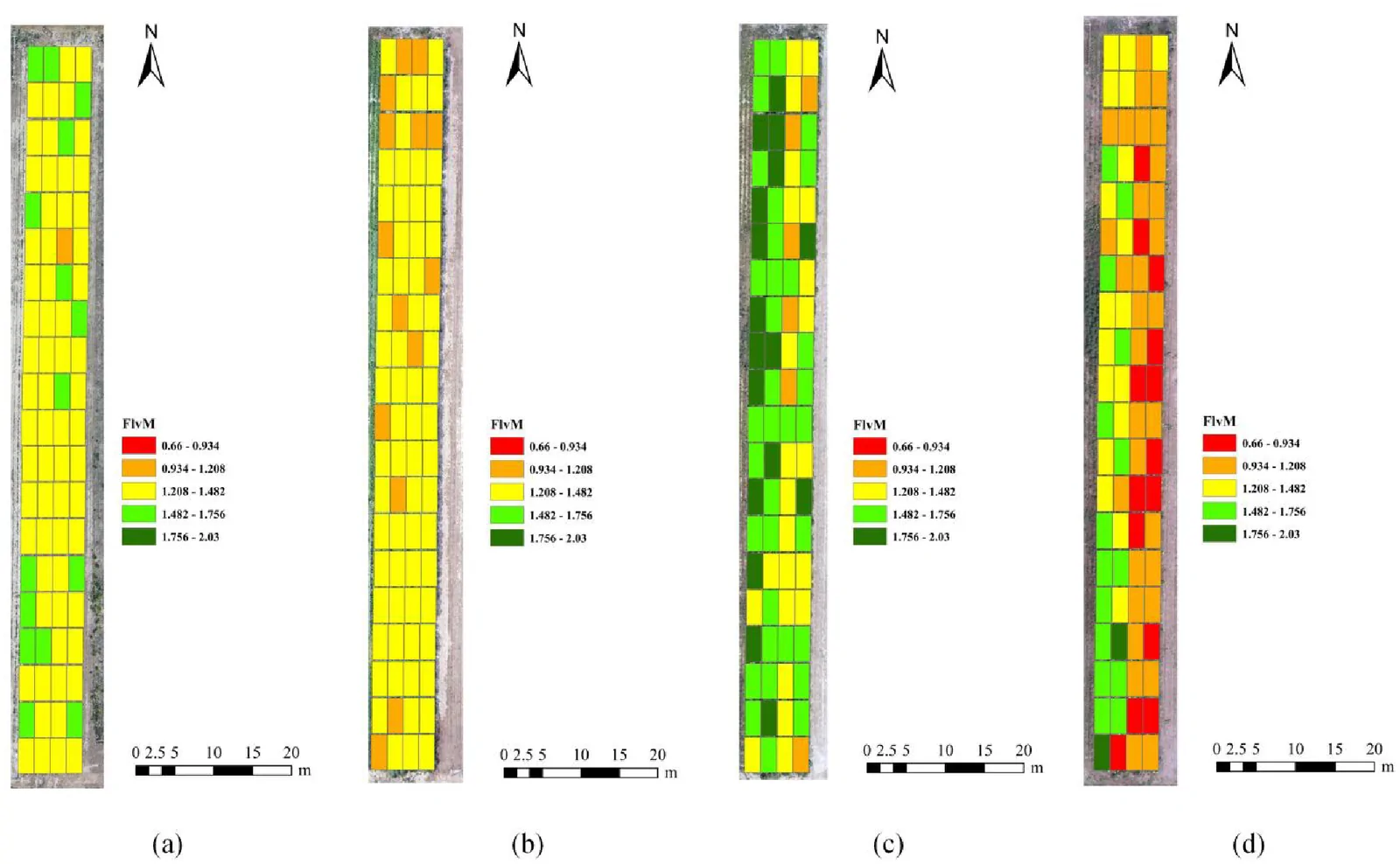
Spatial distribution maps of sunflower leaf Flav across 80 sampling plots generated using the selected GWO-tuned Transformer model with the 50 m dataset. **(a)** Seedling stage; **(b)** budding stage; **(c)** flowering stage; and **(d)** maturity stage. The maps comprise in-sample estimates for the 56 training plots and out-of-sample predictions for the 24 testing plots and are presented for spatial visualization rather than additional model evaluation.

After comparative evaluation, the selected GWO-tuned Transformer model at 50 m was applied to all 80 plots at each growth stage. **Figure 10** includes in-sample estimates for the 56 training plots and out-of-sample predictions for the 24 testing plots and is presented for visualization rather than additional model evaluation. The measured and estimated Flav ranges were 0.99–1.53 and 1.03–1.55 at the seedling stage, 1.02–1.76 and 1.01–1.77 at the budding stage, 0.92–2.05 and 0.93–2.03 at the flowering stage, and 0.68–1.86 and 0.66–1.86 at maturity, respectively. **Figure 11** shows the corresponding spatial patterns. Estimated Flav reached its highest overall level at the flowering stage, whereas the widest estimated range occurred at maturity.

## 4 Discussion

### 4.1 Influence of UAV flight height on sunflower Flav estimation

Among the unoptimized PCC-Boruta-Transformer models, the 30 m model achieved the highest observed testing-set performance, with an R^2^ of 0.932 and an RMSE of 0.060. This result may be associated with the greater native spatial detail available in lower-altitude imagery. UAV flight height affects spatial resolution, image coverage, operational efficiency, and multispectral index values (Stamenković et al., 2024), and consequently influences the representation of crop spectral, textural, and structural information (Zhu et al., 2023). Although all orthomosaics were resampled to a common output resolution, resampling could not recover canopy details that were not captured in the original higher-altitude imagery.

Considering the practical trade-off among image quality, spatial resolution, area coverage, and operational efficiency reported for intermediate flight heights, the 50 m dataset was used as the intermediate-height condition for comparing GA, PSO, and GWO. After GWO tuning, its RMSE decreased to 0.053; however, this improvement reflects both the imagery and hyperparameter optimization. Because the 30 m and 80 m models were not subjected to the same optimization procedure, the optimized 50 m result cannot establish the intrinsic superiority of this flight height.

### 4.2 Effects of feature selection on model performance

Feature selection helped reduce input redundancy and identify informative UAV-derived predictors within the current dataset. PCC and Boruta produced partially overlapping but distinct feature subsets because PCC evaluates the marginal linear association between each feature and Flav, whereas Boruta uses random-forest-based importance to capture nonlinear effects and interactions among variables. Their relative subset sizes and retained variables varied with flight height and feature category. PCC alone may retain variables with strong marginal correlations but limited additional predictive value, whereas Boruta may exclude linearly sensitive features that provide complementary information. The combined PCC-Boruta strategy therefore used the intersection of the two subsets to construct a conservative predictor set supported by both linear association and model-based relevance. Its effect varied among regression algorithms, feature combinations, and flight heights, and PCC or Boruta alone achieved equal or higher testing-set performance in some configurations. Nevertheless, the highest observed testing-set configuration at each flight height used the PCC-Boruta feature subset. The intersection-based strategy was therefore effective for constructing the selected high-performing configurations but should not be interpreted as universally superior to either PCC or Boruta alone. The height-dependent variation in retained features further indicates that feature selection should be adapted to acquisition conditions rather than applying a fixed predictor subset across flight heights.

### 4.3 Contribution of multi-feature fusion to Flav estimation

Among the four evaluated feature combinations, feature set D, which integrated vegetation indices, color features, and texture features, achieved the highest observed overall predictive performance. Vegetation indices remained the dominant predictors because of their sensitivity to canopy spectral response, greenness, pigment status, and physiological variation. Although color and texture features showed weaker predictive ability when used independently, they provided complementary information when combined with vegetation indices. Color features derived from the HSV and LAB spaces characterized variation in canopy visual appearance, whereas texture features extracted from the red-edge and near-infrared bands reflected canopy structural heterogeneity, leaf distribution, and local spatial variation. SHAP analysis further showed that the relative contributions of these feature types varied among flight heights, indicating that their complementary value depended on imaging conditions and canopy representation. These findings support the integration of spectral, color, and texture information for sunflower Flav estimation under the UAV acquisition conditions evaluated in this study.

### 4.4 Observed performance of the transformer model for UAV-based Flav estimation

Among the six regression algorithms, the Transformer achieved the highest observed overall predictive performance across the evaluated flight heights and feature combinations. Its self-attention mechanism may have facilitated adaptive weighting of the input variables and the modeling of nonlinear dependencies among UAV-derived predictors. However, Transformer performance can also be sensitive to input dimensionality, sample size, model initialization, and hyperparameter settings. Its observed advantage should therefore be interpreted as performance under the selected feature subsets, data partition, and training conditions of the present experiment rather than as evidence of universal superiority over KNN, RF, CatBoost, SVR, or LightGBM.

### 4.5 Effect of GWO-based hyperparameter optimization on model performance

At the intermediate flight height of 50 m, GWO-based hyperparameter optimization increased the testing-set R^2^ of the Transformer model from 0.911 to 0.947 and reduced the RMSE from 0.069 to 0.053. Under identical optimization-training and validation partitions, hyperparameter search spaces, population sizes, iteration budgets, and model initialization settings, the GWO-tuned Transformer achieved the highest observed testing-set R^2^ and the lowest testing-set RMSE among the GA-, PSO-, and GWO-tuned models. These results indicate that GWO provided the most favorable hyperparameter configuration among the three optimization methods under the current experimental settings.

The GWO-tuned Transformer should not be interpreted as a newly developed algorithmic framework because GWO was used solely as an established method for Transformer hyperparameter optimization, without modifying either the original GWO updating mechanism or the Transformer architecture. Moreover, each optimization method was evaluated using a single fixed run rather than repeated independent runs. Their relative performance may therefore vary with model initialization, search-space definition, population size, iteration budget, and dataset characteristics. The present results identify the GWO-tuned Transformer as the best observed configuration in this experiment but do not establish the stable, reproducible, or universal superiority of GWO. Repeated independent optimization runs are required to quantify optimization variability, whereas independent multi-year, multi-site, and multi-cultivar datasets are needed to assess model robustness and transferability.

### 4.6 Agronomic implications of sunflower Flav spatial mapping

The spatial distribution maps generated using the selected GWO-tuned Transformer model provided a preliminary visualization of within-field variation in sunflower leaf Flav across the four growth stages. The relatively low and spatially uniform estimates at the seedling stage, the increased levels and heterogeneity during the budding and flowering stages, and the lower overall values but persistent heterogeneity at maturity may be associated with changes in canopy development, pigment status, environmental response, and leaf senescence. These interpretations remain tentative because the underlying physiological processes were not measured directly.

From an agronomic perspective, the maps may support the identification of plots with relatively high, low, or atypical estimated Flav values for subsequent field inspection. However, UAV-derived vegetation indices, color features, and texture features represent indirect spectral, visual, and structural proxies rather than direct optical measurements of Flav. Because these features may also respond to canopy cover, biomass, pigment status, canopy structure, and soil background, the maps should not be interpreted as direct diagnoses of nutrient deficiency, water stress, pests, or diseases. Their potential use for site-specific management therefore requires further field validation.

### 4.7 Limitations and future work

Several limitations should be considered when interpreting the present findings. The experiment was conducted at a single study site during one growing season, over a limited field area of approximately 1,000 m^2^ and under one set of management conditions. Although the testing plots were excluded from model fitting and feature selection, they originated from the same site, growing season, and management environment as the training plots. Moreover, the same testing set was used to compare multiple feature-selection strategies, feature combinations, regression algorithms, flight heights, and optimized models. Consequently, the best reported testing performance may be subject to model-selection optimism and should be interpreted as within-experiment comparative performance rather than as an unbiased estimate of predictive performance in completely unseen environments. Independent multi-year, multi-site, and multi-cultivar datasets are required to evaluate model robustness and transferability.

The internal hyperparameter-optimization procedure also had methodological limitations. The feature subset used for optimization was determined using the complete 56-plot training dataset before it was divided into the 39-plot optimization-training subset and the 17-plot validation subset. Therefore, although plot overlap was prevented, the validation subset was not fully independent of the preceding feature-selection process. Validation RMSE should consequently be regarded as an internal hyperparameter-selection criterion rather than as an independent estimate of model performance. Future studies should adopt nested plot-grouped cross-validation and repeated optimization runs to quantify model and optimization variability.

Uncertainty may also have arisen from canopy segmentation. The accuracy of the NDVI–Otsu-based canopy mask and manual weed exclusion was not evaluated against independently annotated reference masks. Segmentation errors involving sunflower canopy, weeds, shadows, and soil background may therefore have propagated into the extracted vegetation-index, color, and texture features.

The overall R^2^ and RMSE values were calculated by pooling observations from four growth stages. Because the mean and variation of Flav differed among stages, the overall metrics may partly reflect between-stage variation rather than only within-stage differences among plots. Stage-specific performance metrics and leave-one-growth-stage-out validation should therefore be considered in future studies.

Physiological and environmental variables, including chlorophyll content, leaf nitrogen, leaf area, canopy temperature, soil moisture, and nutrient status, were not measured simultaneously. In addition, no controlled irrigation, fertilization, pest-management, disease-management, or yield-response experiments were conducted. These limitations prevented direct determination of the physiological causes of the observed spatial variation and the trait specificity of the selected UAV-derived features. Future research should integrate physiological measurements with intervention-based field experiments before the proposed workflow is used for operational precision management.

## 5 Conclusion

This study evaluated UAV-based RGB–multispectral imagery for estimating sunflower leaf Flav across four growth stages and three flight heights. Vegetation indices were the dominant predictors, whereas color and texture features provided complementary information. Among the unoptimized configurations, the PCC-Boruta-Transformer model using the fully fused feature set D achieved the highest observed predictive performance at 30 m. At the intermediate flight height of 50 m, GWO-based hyperparameter optimization further improved the Transformer model and produced the best observed configuration among all models evaluated in this study. The selected model was subsequently used to visualize within-field variation in sunflower leaf Flav across growth stages.

Overall, the results demonstrate the feasibility of combining RGB–multispectral canopy features, height-specific feature selection, and machine learning for field-scale sunflower Flav estimation under the conditions evaluated in this study. These findings are limited to the specific experimental site, the 2025 growing season, the UAV platforms and sensors, and the crop-management conditions used in this experiment. Independent external validation using multi-year, multi-site, and multi-cultivar datasets is required to evaluate the robustness and transferability of the proposed workflow.

## Data Availability

The raw data supporting the conclusions of this article will be made available by the authors, without undue reservation.
