# Supplementary material for: UAV-based estimation of sunflower leaf flavonol content across multiple flight heights using RGB–multispectral canopy features and machine learning
Source: Front Plant Sci. 2026 Aug 31;17:1931472. doi: 10.3389/fpls.2026.1931472 (PMC13572615; doi:10.3389/fpls.2026.1931472)
Supplement: Supplementary file 1 [file Table1.docx]

Supplementary Table S1 Training performance of sunflower leaf Flav estimation models at a flight height of 30 m

| **Algorithm** | **Feature set** | **PCC** | | **Boruta** | | $\mathbf{PCC}$**-Boruta** | |
| --- | --- | --- | --- | --- | --- | --- | --- |
|  |  | **R^2^** | **RMSE** | **R^2^** | **RMSE** | **R^2^** | **RMSE** |
| KNN | A | 0.694 | 0.136 | 0.712 | 0.132 | 0.717 | 0.131 |
|  | B | 0.594 | 0.157 | 0.504 | 0.173 | 0.465 | 0.180 |
|  | C | 0.676 | 0.140 | 0.720 | 0.130 | 0.733 | 0.127 |
|  | D | 0.717 | 0.131 | 0.729 | 0.128 | 0.748 | 0.124 |
| RF | A | 0.679 | 0.139 | 0.726 | 0.129 | 0.718 | 0.131 |
|  | B | 0.482 | 0.177 | 0.528 | 0.169 | 0.591 | 0.157 |
|  | C | 0.680 | 0.139 | 0.722 | 0.130 | 0.763 | 0.120 |
|  | D | 0.719 | 0.130 | 0.766 | 0.119 | 0.801 | 0.110 |
| Transformer | A | 0.896 | 0.079 | 0.840 | 0.098 | 0.912 | 0.073 |
|  | B | 0.643 | 0.147 | 0.534 | 0.168 | 0.573 | 0.161 |
|  | C | 0.907 | 0.075 | 0.810 | 0.107 | 0.924 | 0.068 |
|  | **D** | **0.938** | **0.061** | **0.943** | **0.059** | **0.940** | **0.060** |
| CatBoost | A | 0.726 | 0.129 | 0.752 | 0.123 | 0.853 | 0.094 |
|  | B | 0.589 | 0.158 | 0.639 | 0.148 | 0.686 | 0.138 |
|  | C | 0.755 | 0.122 | 0.797 | 0.111 | 0.784 | 0.114 |
|  | D | 0.781 | 0.115 | 0.803 | 0.109 | 0.921 | 0.069 |
| SVR | A | 0.778 | 0.116 | 0.652 | 0.145 | 0.666 | 0.142 |
|  | B | 0.592 | 0.157 | 0.470 | 0.179 | 0.634 | 0.149 |
|  | C | 0.710 | 0.132 | 0.685 | 0.138 | 0.740 | 0.125 |
|  | D | 0.877 | 0.086 | 0.689 | 0.137 | 0.776 | 0.116 |
| LightGBM | A | 0.711 | 0.132 | 0.701 | 0.135 | 0.825 | 0.103 |
|  | B | 0.725 | 0.129 | 0.745 | 0.124 | 0.753 | 0.122 |
|  | C | 0.750 | 0.123 | 0.720 | 0.130 | 0.766 | 0.119 |
|  | D | 0.820 | 0.104 | 0.785 | 0.114 | 0.839 | 0.099 |
