# Supplementary material for: UAV-based estimation of sunflower leaf flavonol content across multiple flight heights using RGB–multispectral canopy features and machine learning
Source: Front Plant Sci. 2026 Aug 31;17:1931472. doi: 10.3389/fpls.2026.1931472 (PMC13572615; doi:10.3389/fpls.2026.1931472)
Supplement: Supplementary file 2 [file Table2.docx]

Supplementary Table S2 Training performance of sunflower leaf Flav estimation models at a flight height of 50 m

| **Algorithm** | **Feature set** | **PCC** | | **Boruta** | | $\mathbf{PCC}$**-Boruta** | |
| --- | --- | --- | --- | --- | --- | --- | --- |
|  |  | **R^2^** | **RMSE** | **R^2^** | **RMSE** | **R^2^** | **RMSE** |
| KNN | A | 0.680 | 0.139 | 0.617 | 0.152 | 0.715 | 0.131 |
|  | B | 0.318 | 0.203 | 0.483 | 0.177 | 0.551 | 0.165 |
|  | C | 0.613 | 0.153 | 0.697 | 0.135 | 0.613 | 0.153 |
|  | D | 0.656 | 0.144 | 0.734 | 0.127 | 0.620 | 0.152 |
| RF | A | 0.634 | 0.149 | 0.702 | 0.134 | 0.679 | 0.139 |
|  | B | 0.432 | 0.185 | 0.585 | 0.159 | 0.558 | 0.164 |
|  | C | 0.680 | 0.139 | 0.713 | 0.132 | 0.699 | 0.135 |
|  | D | 0.710 | 0.132 | 0.725 | 0.129 | 0.737 | 0.126 |
| Transformer | A | 0.892 | 0.081 | 0.737 | 0.126 | 0.914 | 0.072 |
|  | B | 0.501 | 0.174 | 0.518 | 0.171 | 0.634 | 0.149 |
|  | C | 0.873 | 0.088 | 0.758 | 0.121 | 0.915 | 0.072 |
|  | **D** | **0.878** | **0.086** | **0.889** | **0.082** | **0.939** | **0.061** |
| CatBoost | A | 0.759 | 0.121 | 0.801 | 0.110 | 0.850 | 0.095 |
|  | B | 0.683 | 0.139 | 0.715 | 0.131 | 0.720 | 0.130 |
|  | C | 0.781 | 0.115 | 0.821 | 0.104 | 0.805 | 0.109 |
|  | D | 0.805 | 0.109 | 0.852 | 0.095 | 0.882 | 0.085 |
| SVR | A | 0.666 | 0.142 | 0.614 | 0.153 | 0.617 | 0.152 |
|  | B | 0.589 | 0.158 | 0.542 | 0.167 | 0.551 | 0.165 |
|  | C | 0.700 | 0.135 | 0.620 | 0.152 | 0.613 | 0.153 |
|  | D | 0.719 | 0.130 | 0.644 | 0.147 | 0.620 | 0.152 |
| LightGBM | A | 0.650 | 0.146 | 0.690 | 0.137 | 0.725 | 0.129 |
|  | B | 0.553 | 0.165 | 0.580 | 0.159 | 0.620 | 0.152 |
|  | C | 0.680 | 0.139 | 0.730 | 0.128 | 0.750 | 0.123 |
|  | D | 0.700 | 0.135 | 0.760 | 0.121 | 0.788 | 0.113 |
