# Supplementary material for: UAV-based estimation of sunflower leaf flavonol content across multiple flight heights using RGB–multispectral canopy features and machine learning
Source: Front Plant Sci. 2026 Aug 31;17:1931472. doi: 10.3389/fpls.2026.1931472 (PMC13572615; doi:10.3389/fpls.2026.1931472)
Supplement: Supplementary file 3 [file Table3.docx]

Supplementary Table S3 Training performance of sunflower leaf Flav estimation models at a flight height of 80 m

| **Algorithm** | **Feature set** | **PCC** | | **Boruta** | | $\mathbf{PCC}$**-Boruta** | |
| --- | --- | --- | --- | --- | --- | --- | --- |
|  |  | **R^2^** | **RMSE** | **R^2^** | **RMSE** | **R^2^** | **RMSE** |
| KNN | A | 0.671 | 0.141 | 0.668 | 0.142 | 0.668 | 0.142 |
|  | B | 0.334 | 0.201 | 0.506 | 0.173 | 0.531 | 0.169 |
|  | C | 0.579 | 0.160 | 0.693 | 0.136 | 0.703 | 0.134 |
|  | D | 0.630 | 0.150 | 0.700 | 0.135 | 0.733 | 0.127 |
| RF | A | 0.652 | 0.145 | 0.693 | 0.136 | 0.679 | 0.139 |
|  | B | 0.503 | 0.173 | 0.492 | 0.175 | 0.551 | 0.165 |
|  | C | 0.610 | 0.154 | 0.699 | 0.135 | 0.698 | 0.135 |
|  | D | 0.705 | 0.134 | 0.724 | 0.129 | 0.737 | 0.126 |
| Transformer | A | 0.841 | 0.098 | 0.866 | 0.090 | 0.906 | 0.075 |
|  | B | 0.367 | 0.196 | 0.531 | 0.169 | 0.536 | 0.168 |
|  | C | 0.842 | 0.098 | 0.888 | 0.082 | 0.865 | 0.090 |
|  | **D** | **0.847** | **0.096** | **0.935** | **0.063** | **0.913** | **0.073** |
| CatBoost | A | 0.711 | 0.132 | 0.751 | 0.123 | 0.780 | 0.115 |
|  | B | 0.618 | 0.152 | 0.631 | 0.149 | 0.650 | 0.146 |
|  | C | 0.740 | 0.125 | 0.760 | 0.121 | 0.750 | 0.123 |
|  | D | 0.800 | 0.110 | 0.830 | 0.101 | 0.850 | 0.095 |
| SVR | A | 0.667 | 0.142 | 0.604 | 0.155 | 0.683 | 0.139 |
|  | B | 0.436 | 0.185 | 0.372 | 0.195 | 0.442 | 0.184 |
|  | C | 0.631 | 0.149 | 0.635 | 0.149 | 0.687 | 0.138 |
|  | D | 0.757 | 0.121 | 0.639 | 0.148 | 0.711 | 0.132 |
| LightGBM | A | 0.660 | 0.143 | 0.700 | 0.135 | 0.725 | 0.129 |
|  | B | 0.480 | 0.177 | 0.520 | 0.170 | 0.550 | 0.165 |
|  | C | 0.690 | 0.137 | 0.720 | 0.130 | 0.700 | 0.135 |
|  | D | 0.720 | 0.130 | 0.780 | 0.115 | 0.801 | 0.110 |
